# The nature of protein intake as a discriminating factor of diet sustainability: a multi-criteria approach

**DOI:** 10.1038/s41598-023-44872-3

**Published:** 2023-10-19

**Authors:** Hafsa Toujgani, Joséphine Brunin, Elie Perraud, Benjamin Allès, Mathilde Touvier, Denis Lairon, François Mariotti, Philippe Pointereau, Julia Baudry, Emmanuelle Kesse-Guyot

**Affiliations:** 1 Université Sorbonne Paris Nord and Université Paris Cité, Inserm, INRAE, CNAM, Center of Research in Epidemiology and StatisticS (CRESS), Nutritional Epidemiology Research Team (EREN), 74 Rue Marcel Cachin, 93017 Bobigny, France; 2 ADEME, (Agence de l’Environnement et de la Maîtrise de l’Energie), 49004 Angers, France; 3 Université Paris-Saclay, AgroParisTech, INRAE, UMR PNCA, 91120 Palaiseau, France; 4 Aix Marseille Université, Inserm, INRAE, C2VN, 13005 Marseille, France; 5 Solagro, 75, Voie TOEC, CS 27608, 31076 Toulouse Cedex 3, France

**Keywords:** Environmental impact, Sustainability

## Abstract

Animal production is responsible for 56–58% of the GHG emissions and limiting meat consumption would strongly contribute to reducing human health risks in Western countries. This study aimed to investigate the nature of protein intake as a discriminating factor for diets’ sustainability. Using data from 29,210 French adults involved in the NutriNet-Santé cohort, we identified clusters according to 23 protein sources. A multicriteria (environmental, economic, nutritional and health) sustainability analysis was then conducted on the identified clusters. The economic analysis focused on both food and protein expenditure structures, using a budget coefficient approach. Relative values of clusters compared to the whole sample were calculated. We identified five clusters: milk-based, meat-based, fast food-based, healthy-fish-based, and healthy-plant-based. We found that the healthy-plant-based and healthy-fish-based clusters were the most sustainable, conciliating the compromise between human health (0.25 and 0.53 respectively for the Health Risk Score) and the protection of the environment (− 62% and − 19% respectively for the pReCiPe indicator). Conversely, the highest environmental impacts (+ 33% for the pReCiPe indicator) and the highest health risk (0.95 for the HRS) were observed for the meat-based cluster, which was associated with the lowest nutritional scores (− 61% for the PNNS-GS2 score). The economic analysis showed that the healthy-plant-based cluster was the one with the highest food budget coefficient (+ 46%), followed by the healthy-fish-based cluster (+ 8%), partly explained by a strong share of organic food in the diet. However, the meat-based cluster spent more of their food budget on their protein intake (+ 13%), while the healthy-plant-based cluster exhibited the lowest expenditure for this intake (− 41%). Our results demonstrate that the nature of protein intake is a discriminating factor in diet sustainability. Also, reducing animal protein consumption would generate co-benefits beyond environmental impacts, by being favorable for health, while reducing the monetary cost associated with protein intake.

## Introduction

Some planetary boundaries being officially crossed^1–3^, recent efforts to limit global warming to 1.5 °C, as stipulated in the Paris Climate Agreement, remain insufficient according to the 2021 Intergovernmental Panel on Climate Change (IPCC) report^4^. Moreover, the 2023 Sustainable Development Goals Report not only confirms this assessment but also sounds the alarm, urgently calling for a doubling of efforts to get these objectives back on track^5^. Actually, current food systems are responsible for one third of global GHG emissions^6^, one third to 80% of which originates from the production stage^6,7^. Besides, as populations become more urbanized and affluent, their dietary patterns are shifting towards calorie-rich diets with more animal-based and protein-dense foods^8^. These dietary patterns have been largely influenced by the “protein” debate, which has been evolving for decades. It was in the 1930s that particular attention was paid to this nutrient, with the widespread incidence of kwashiorkor, and its association with protein deficiency^9^. A special Protein Advisory Group was then created by the United Nations, whose mission was to “fight to close the protein gap”, while dietary guidelines encouraged the consumption of protein-rich foods, namely meat and milk^10^. Furthermore, the “protein” debate has been largely influenced by animal production industry, given the great market opportunities offered by this sector, whose trade balance reached 28 billion euros in 2018 in the EU^11^. However, the debate has gone off in another direction, and it is now beyond doubt that the reduction of meat consumption would contribute to strongly reducing human health risks in Western countries according to the Global Burden of Diseases (GBD) analysis^12^. Moreover, animal food production is responsible for 56–58% of the emissions generated by food production while providing only 37% of the protein supply^13^. In that regard, the IPCC has strongly recommended to reduce meat consumption by two-thirds^4^, as red meat and processed meat production have been shown to have the highest impact on all dimensions (GHG emissions, land use, water use, acidification and eutrophication)^13,14^. Note that these emissions are double those generated by plant-based foods^15^. Although it has been proven that there is no longer protein gap in Western countries, as protein intake exceeding needs^16^, the protein debate persists and has shifted towards new trends of “protein transitions”. The new markets of plant-based meat and dairy substitutes are growing exponentially^17^, contributing to collapse the global dietary issues into a single nutrient. That being said, the individuals’ dietary patterns seem to be strongly influenced by this debate. Indeed, it has been shown that the overall diet of meat eaters is less healthy than the one of plant-based foods eaters^18–20^.

Thus, as we assume that the nature of protein intake might be indicative of the overall dietary patterns of individuals, we hypothesized that it could be a discriminating factor for diet sustainability, as defined by FAO in 2012^21^. We identified a previous study comparing diets defined by protein sources in relation to sustainability^22^ but the economic dimension was not assessed. However, we believe that this aspect holds considerable importance regarding individual dietary choices, particularly given the established literature linking healthier and acceptable diets to increased expenses^23^. Our objective was to identify clusters in the population of French adults participating in the NutriNet-Santé cohort, according to the sources of protein intake, and then to characterize, in a multi-criteria approach, the level of sustainability of these clusters according to environmental, economic, nutritional and health aspects. We specifically conducted an economic analysis to investigate the expenditure structure of both food and protein intake across the identified clusters as this dimension of diets’ sustainability is often omitted.

## Methods and data

### Study population

The present study used observational data from the NutriNet-Santé study. The NutriNet-Santé study is an Internet-based cohort launched in May 2009^24^. Its purpose is to study the determinants of diets, nutritional status, and physical activity as well as their associations with health. The participants, recruited on a voluntary basis, are adults living in France with an access to internet. Participants have to complete annual or biannual questionnaires on socioeconomic status, lifestyle, anthropometry, dietary intake and physical activity. Regularly, specific questionnaires are proposed. Gender, occupational status, income, place of residence, physical activity, and smoking habits are self-reported using validated questionnaires^25^. The NutriNet- Santé study is in line with the principles of the Helsinki Declaration^26^ and the protocol has been approved by both the INSERM Ethical Evaluation Committee (CEEI) (no. 0000388FWA00005831) and the National Committee for Information Technology and Freedom (CNIL) (nos. 908450 and 909216). Informed consent was obtained from all participants. The study is registered in ClinicalTrials.gov (NCT03335644).

### Assessment of food consumption and protein intake in total and by food groups

**Figure 1:**
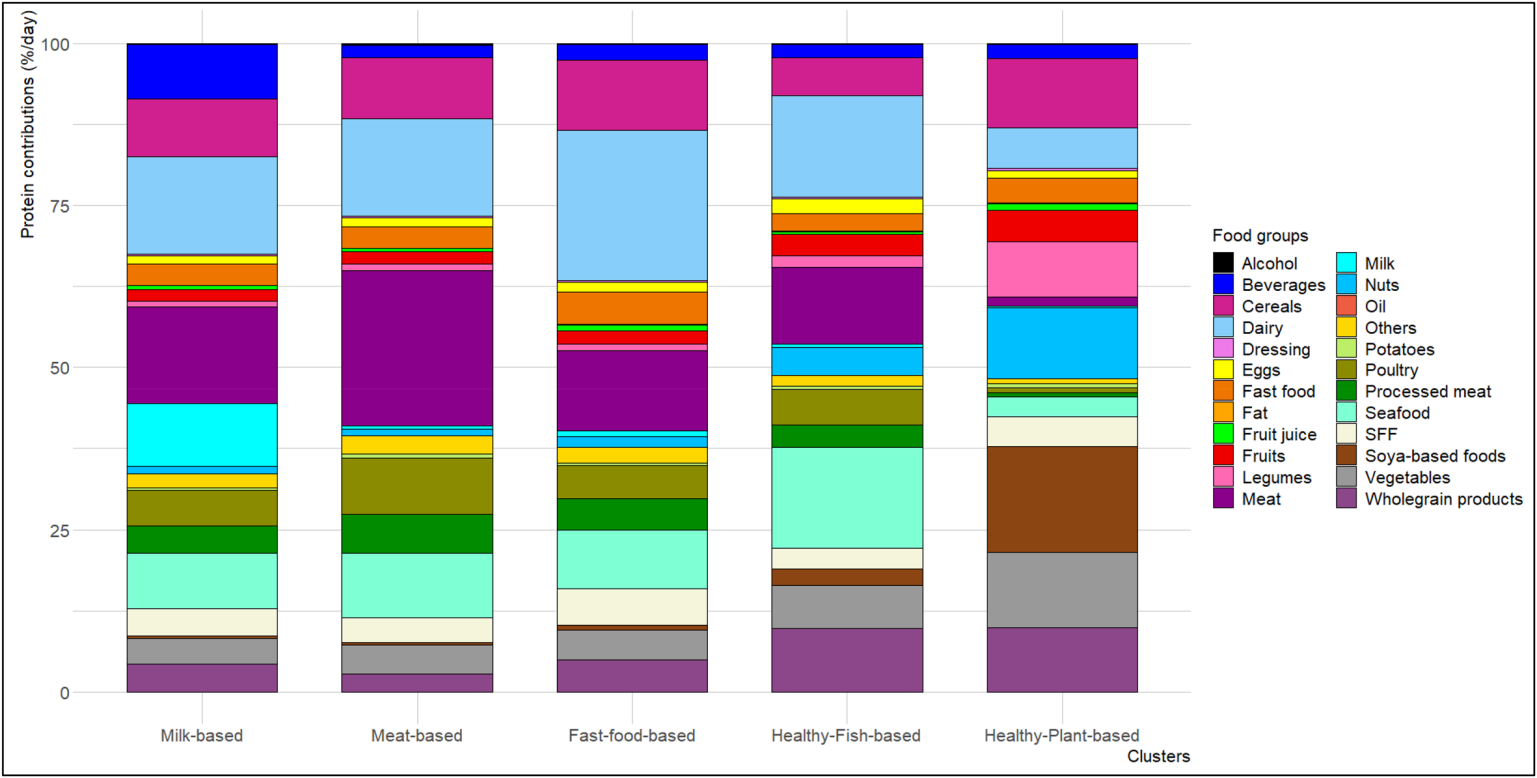
**Protein contributions per food group across clusters**. Values are energy-adjusted means of protein contributions of food groups (%/day) computed using ANCOVA model. Food groups are formed as follows: Vegetables include all vegetables and soups; Fruits include fresh fruits, fruits in syrup and compote, dried fruits and seeds; Beverages include all non-alcoholic beverages that are fruit nectar, syrup, soda (with or without sugar), plant-based beverages (except soya-based), milk consumed with tea/coffee; Dairy products include yogurts, fresh cheese and cheese; Potatoes include other tubers; Cereals include breakfast cereal low in sugar, bread semolina, rice and pasta; SFF (sweet and fat foods) include croissants, pastries, chocolate, biscuits, milky dessert, ice cream, honey and marmalade, cakes, chips, salted oilseeds, salted biscuits; Fast-food include sandwich, prepared foods such as pizza, hamburger, ravioli, panini, salted pancake, etc.; Soya-based food includes tofu, soya-based meat substitute and vegetable patties, soya-based yogurt, soya-based milk; Fat includes animal fat (butter and lards); Dressing includes ready-to-use salad dressing, mayonnaise or cream-based sauces, sour cream and butter and all fat-based sauces; Oil includes plant-based oils; Meat includes beef and pork.

Food consumption data were collected via an Organic Food Frequency Questionnaire (Org-FFQ) developed in 2014, including 264 organic and conventional food items^27^. In the present study, a total of 23 food groups were defined based on their protein content as follows: meat (including beef and pork), processed meat, poultry, seafood, eggs, milk, dairy (including all dairy products except for milk), fast food, sweetened and fatty foods (SFF), fat (including animal fat and margarine), dressing, potatoes, legumes, whole-grain products, cereals (including all cereals products), nuts, soya-based products (including also substitutes), vegetables, fruits, fruit juice, beverages (including all non-alcoholic beverages), oil (including vegetable oils) and alcohol (including all alcoholic beverages). Nutrient values were derived from the food composition table developed for the NutriNet-Santé study^28^. Detailed information on the composition of the food groups is provided in the legend of Fig. 1.

### Environmental data

Environmental pressures, greenhouse gas (GHG) emissions (CO2-eq), Cumulative Energy Demand (MJ), Land use (m^2^), were assessed using the DIALECTE tool^29^. Developed by Solagro, this French diagnostic tool aims to evaluate the environmental performance of French farms using a comprehensive approach. The Life Cycle Assessment method was used on 60 raw agricultural products. The scope of the analysis was limited to the agricultural production stage but organic and conventional products were distinguished. Details are provided in Supplemental Material 1. In addition, the pReCiPe score, a synthetic impact indicator including the three indicators above, has been calculated^30^. To balance conflicting environmental indicators, the ReCiPe method considers both midpoint and endpoint measures. Developed in the Netherlands, this LCA method aligns the indicators to provide a comprehensive view^30^. It focuses on 18 indicators, three of which are oriented towards final impacts, including resource availability, human health and ecosystem diversity. In practice, some authors have found that the environmental impact of food products and diets can be assessed by measuring greenhouse gas emissions, primary energy consumption, and land occupation. These factors make up about 90% of the total environmental dimension of the ReCiPe model. To calculate the environmental impact of a food product or diet, one can use the partial ReCiPe score (pReCiPe), with normalization and weighting factors^31^.

The pReCiPe score is computed as follows:

$$pReCiPe = 0.0459 \times GHGe + 0.0025 \times CED + 0.0439 \times LO$$

with GHGe, in kg of CO_2_eq/d, CED, in MJ/d and LO, in m^2^/d. The highest the pReCiPe, the highest the environmental impact.

### Nutritional quality data

Three dietary indexes were computed. The PANDiet (Diet Quality Index based on the Probability of Adequate Nutrient Intake) is a nutritional adequacy score based on the nutritional references values^32,33^. The PNNS-GS2 (Programme National Nutrition Santé-Guidelines Score 2) measures the adherence of individuals to the French dietary guidelines established by the High Council of Public Health in 2017^34^. The cDQI (Comprehensive Diet Quality Index) aims to assess the quality of plant and animal foods consumed^35^. Further details are provided in Supplemental Material 2.

### Health risk data

Health risk was assessed using a “*Health Risk Score*” (HRS) of the diet, computed using the distance to the Theoretical Minimum-Risk Exposure Level (TMREL), provided in the GBD study in 2019^12^. It reflects the overall risk of death associated with the individual dietary pattern, resulted from a suboptimal consumption of each food group. The computation of the HRS, ranging from 0 to 1, is provided in the Supplemental Material 3.

### Economic data

The economic data used were participants' monthly income, and their estimated food expenditures for their whole diet and each food group.

Participants' income was collected as part of the socio-economic status questionnaire, where each participant provided the income class corresponding to his/her monthly income. Income per consumption unit (C.U) were estimated using household composition and age of family members according to the INSEE procedure^36^. In the NutriNet-Santé study, the monetary cost of the diet (€/d) was calculated for each participant using prices (€/g) from several databases. Further details are provided in Supplemental Material 4.

### Statistical analysis

Among the participants in the cohort NutriNet-Santé, 29, 210 individuals were selected for this study, with Org-FFQ data, no missing data for sociodemographic aspects (except for monthly income which is a non-mandatory question) and with available information on place of purchase. Those considered as under- or over-reporters for energy intake were excluded as previously published^27^. A flowchart is provided in Supplemental Figure 1.

### Construction of the protein-source-typology and description of clusters

The contribution (in %) to total protein intake of the 23 food groups was calculated for each individual, to focus on the sources independently of the total intake. The typology aiming to identify groups of individuals with similar protein sources was built using a two-step procedure. First, a Principal Component Analysis^37^ was applied on the 23 protein contributing food groups (the list is available on Fig. 1). This multivariate data analysis method allowed to reduce the initial range of information by maximizing the variance. Nine dimensions were retained according to Kaiser criterion (eigenvalues > 1). Then, on the basis of the retained dimensions, an Ascending Hierarchical Classification (AHC) was performed with data preprocessing using the K-means algorithm reiterated 100 times. As this study used a large database, the complementary use of the k-means and AHC methods allowed to stabilize the solution. Further details are provided in Supplemental Material 5.

### Description and comparison of clusters

The clusters were named according to the food groups contributing the most to the protein intake of each cluster compared to the whole sample. First, means (SD) of protein contribution of each food group were computed (%/day) for the whole sample. Then, as cluster potentially exhibits a different energy intake than the whole sample, energy-adjusted means of protein contributions of the 23 food groups (SEM) were calculated for each cluster, using ANCOVA models.

The identified clusters were described according to the socio-demographic characteristics reporting mean (SD) or % for continuous and categorical variables respectively. Means comparison across clusters was performed using Pearson’s Chi-square test for categorical variables and ANOVA test for continuous variables. For food groups consumption, mean (SD) were presented for the whole sample, and for each cluster, energy-adjusted mean of food group intakes (g/day) and standard error of the mean (SEM) were calculated, using ANCOVA models.

Percentages of total energy intake were calculated for macronutrients. For vitamins, minerals and fiber, each nutrient energy-adjusted intake was calculated based on the “residual method”^38^. Prevalence of adequate protein intake is computed as defined in the PANDiet score^33^.

To allow comparison of clusters to the whole sample in relative values for all indicators, standardized means were computed for the whole sample, corresponding to the mean that the whole sample would have if its energy intake was that of the cluster (${{\varvec{o}}{\varvec{v}}{\varvec{e}}{\varvec{r}}{\varvec{a}}{\varvec{l}}{\varvec{l}}}_{{\varvec{i}}})$. Relative values as regards energy-adjusted indicator, were then calculated with the following formula:

$${\rm{Relative\, value \,of \,indicator}} _{i} \left( \% \right) = \frac{{ {\rm{Energy\, adjusted\, mean}}_{i} - {\rm{Standardized\, mean}}_{{overall_{i} }} }}{{\rm{Standardized \,mean}_{{overall_{i} }} }} \times 100$$

where i denotes clusters.

#### Multicriteria analysis

For each sustainability indicator considered, we calculated the mean (SD), and for each cluster, energy-adjusted means (SEM) were calculated via ANCOVA models. Comparison between clusters was based on relative values computed as defined above. A comparison of means across clusters was performed using ANCOVA models.

### Economic analysis

The objective was to analyze both food and protein expenditure structure across clusters. The economic analysis included 27,244 of the 29,210 participants, for whom there were no missing income data (since the question was optional). The monthly income variable, modelled, as category was transformed into a numeric variable by considering the class center of the daily income category for each individual as previously done^39^ and converted as euros per day.

The expenditure structure analysis across clusters was conducted using a budget coefficient approach^40^. This approach makes comparable the share of food expenditure between individuals with different incomes and different diets^40^.

To do so, we first computed for each participant, the budget coefficients of both the overall diet and the food groups, using the following formulas:

$$\begin{aligned} & {\rm{Budget \,coefficient \,of \,the \,overall \,diet_{i}}} = \frac{\rm{Overall \,diet \,expenditure_{i} }}{{Income_{i} }} \times 100 \\ & {\rm{Budget \,coefficient \,of \,food \,group_{i,j}}} = \frac{\rm{Food \,group \,expenditure_{i,j} }}{\rm{Overall \,diet \,expenditure_{i} }} \times 100 \\ \end{aligned}$$

where i denotes individuals and j denotes food groups.

Insofar as we assume that the production mode (organic/conventional) affects food expenditure, the analysis was detailed by distinguishing expenditures allocated to organic products from those allocated to conventional products. To do so, budget coefficients of organic and conventional foods, for the overall diet and for each food group were computed for each individual. For the overall diet, budget coefficients of the overall diet by production mode were computed with respect to the overall diet budget. The budget coefficients of the food groups by production mode were calculated in relation to the overall diet budget allocated to foods from the corresponding production mode.

We defined the protein expenditure as the share of the food group expenditure allocated to the daily protein intake. It was calculated, for each food group and for each participant, using the following formula:

$${\text{Protein expenditure}}_{i} ,_{j} \left( euro \right) = \frac{{~{\text{Food expenditure}}_{i} ,_{j} ~\left( euro \right) \times ~{\text{Protein intake}}_{i} ,_{j} ~\left( g \right)}}{{{\text{Quantity consumed}}_{i} ,_{j} ~\left( g \right)~}}$$

where i denotes individuals and j denotes food groups.

Then, the “*Total protein expenditure*” was calculated for each participant by summing the protein expenditures for all food groups.

The budget coefficients of protein intake were then computed, using the following formulas:

$$\begin{aligned} & {\rm{Budget \;coefficient \;of \;protein}}\; {\text{intake}} {\rm{\; per\; food \;group_{i,j}}} = \frac{\rm{Protein \;expenditure_{i,j} }}{\rm{Overall \;diet \;expenditure_{i} }} \times 100 \\ & {\rm{Budget \;coefficient\; of\; the \;total\; protein}}\; {\text{int}} ake_{i} = \frac{\rm{Total\; Protein\; expenditure_{i} }}{\rm{Overall \;diet\; expenditure_{i} }} \times 100 \\ \end{aligned}$$

where i denotes individuals and j denotes food groups.

Afterwards, non-adjusted means (SD) were computed for all the calculated budget coefficients for the whole sample, and for each cluster, means and standard error of the mean (SEM) adjusted for energy intake were estimated using ANCOVA models. Comparison between clusters was based on relative percentage values computed using standardized means as defined above. Comparison of means across clusters was performed using the ANCOVA test.

Data management and statistical analyses were performed using RStudio software (RStudio, Version 1.4.1717, © 2009–2021 RStudio, PBC).

## Results

The study population was predominantly female (75%), with a mean age of 54 (SD = 14) years (Supplemental Table 1).

### Description of the protein-source typology

Based on food group contribution to protein intake, we identified 5 clusters (Fig. 1 and Table 1): *Milk-based* cluster (17% of the population), characterized by high contributions of milk (+ 336% higher than the whole sample) and beverages (coffee, tea (including with milk), all sweetened beverages except fruit juice 100%); *Meat-based* cluster (26% of the population) with high contributions of red meat to proteins (+ 54%), poultry and processed meat; *Fast-food-based* cluster (29% of the population) showing a higher protein intake derived from fast food to proteins (+ 37%), refined or wholegrain cereals, fatty and sweet products; *Healthy-fish-based* cluster (25% of the population), characterized by high protein intake from seafood (+ 49%), wholegrain cereal products, fruit and vegetables; and finally the *Healthy-plant-based* cluster (3% of the population) for which the most of protein intake is derived from the consumption of soy (+ 909%), legumes, nuts, fruit and vegetables. The detailed values are presented in Table 1, and Fig. 1.

**Table 1:** Protein contributions of food groups across clusters.

Food groups	Whole sample	Milk-based	Meat-based	Fast-food-based	Healthy-fish-based	Healthy-plant-based	*p*
	n = 29,210	n = 4966 (17%)	n = 7569 (26%)	n = 8469 (29%)	n = 7189 (25%)	n = 1017 (3%)	
Legumes	1.41 (2.49)	0.86 (0.03)	0.95 (0.02)	1.06 (0.02)	1.71 (0.02)	8.49 (0.07)	< 0.0001
Soya-based foods	1.62 (4.72)	0.43 (0.05)	0.42 (0.04)	0.83 (0.04)	2.56 (0.04)	16.33 (0.12)	< 0.0001
Cereals	8.9 (6.38)	8.83 (0.09)	9.39 (0.07)	10.84 (0.07)	5.91 (0.07)	10.68 (0.19)	< 0.0001
Nuts	2.39 (4.37)	1.23 (0.05)	1.00 (0.04)	1.60 (0.04)	4.37 (0.04)	11.00 (0.12)	< 0.0001
Wholegrain products	5.59 (7.06)	4.26 (0.09)	2.74 (0.07)	4.91 (0.07)	9.73 (0.08)	9.93 (0.20)	< 0.0001
Fruits	2.41 (2.69)	1.83 (0.04)	1.98 (0.03)	2.07 (0.03)	3.35 (0.03)	4.81 (0.08)	< 0.0001
Fruit juice	0.61 (0.91)	0.59 (0.01)	0.44 (0.01)	0.89 (0.01)	0.42 (0.01)	1.07 (0.03)	< 0.0001
Vegetables	5.21 (3.63)	4.00 (0.05)	4.45 (0.04)	4.61 (0.04)	6.69 (0.04)	11.55 (0.10)	< 0.0001
Potatoes	0.46 (0.49)	0.39 (0.01)	0.59 (0.01)	0.42 (0.01)	0.41 (0.01)	0.60 (0.01)	< 0.0001
Alcohol	0.22 (0.53)	0.16 (0.01)	0.29 (0.01)	0.22 (0.01)	0.21 (0.01)	0.20 (0.01)	< 0.0001
Dressing	0.23 (0.28)	0.18 (0.00)	0.21 (0.00)	0.31 (0.00)	0.21 (0.00)	0.31 (0.01)	< 0.0001
Beverages	3.19 (4.24)	8.46 (0.05)	1.99 (0.04)	2.38 (0.04)	1.95 (0.04)	2.13 (0.11)	< 0.0001
Oil	0 (0.00)	0.00 (0.00)	0.00 (0.00)	0.00 (0.00)	0.00 (0.00)	0.00 (0.00)	< 0.0001
Meat	15.26 (10.16)	14.86 (0.12)	23.94 (0.10)	12.36 (0.10)	11.8 (0.10)	1.35 (0.26)	< 0.0001
Processed meat	4.55 (3.88)	4.22 (0.05)	5.99 (0.04)	4.84 (0.04)	3.50 (0.04)	0.55 (0.12)	< 0.0001
Eggs	1.6 (1.66)	1.34 (0.02)	1.36 (0.02)	1.47 (0.02)	2.25 (0.02)	1.23 (0.05)	< 0.0001
Fast food	3.64 (3.24)	3.22 (0.04)	3.31 (0.04)	4.94 (0.03)	2.73 (0.04)	3.81 (0.10)	< 0.0001
Fat	0.08 (0.13)	0.07 (0.00)	0.07 (0.00)	0.12 (0.00)	0.08 (0.00)	0.12 (0.00)	< 0.0001
Poultry	6.03 (5.14)	5.38 (0.07)	8.76 (0.05)	5.03 (0.05)	5.52 (0.06)	0.83 (0.15)	< 0.0001
Dairy	17.28 (10.06)	15.14 (0.13)	15.05 (0.11)	23.16 (0.10)	15.73 (0.11)	6.29 (0.29)	< 0.0001
Seafood	10.55 (7.84)	8.51 (0.10)	9.90 (0.08)	9.09 (0.08)	15.42 (0.08)	3.14 (0.23)	< 0.0001
Milk	2.19 (5.06)	9.68 (0.05)	0.57 (0.04)	0.87 (0.04)	0.57 (0.04)	0.31 (0.12)	< 0.0001
SFF	4.24 (3.42)	4.20 (0.05)	3.81 (0.04)	5.52 (0.04)	3.21 (0.04)	4.53 (0.10)	< 0.0001

Values are mean (SD) contribution (%) for the whole sample, and energy-adjusted means of protein contributions (SEM) across clusters (ANCOVA model).

*P* values were calculated using ANCOVA.

Other food groups with minor contribution to protein intake are not represented.

*SFF* sweetened and fatty foods.

The characteristics of the participants in the 5 clusters are shown in Supplemental Table 1. Food group consumptions across clusters are presented in Supplemental Figure 2, and computed nutrient intakes across clusters are presented in Supplemental Table 2. Total Protein intakes range from 67 g/d in the healthy-plant-based cluster to 99 g/d in the meat-based cluster, while plant-based protein intakes vary from 25 g/d in the meat-based cluster to 53 g/d in the healthy-plant-based cluster.

### Multi-criteria analysis of clusters

**Figure 2:**
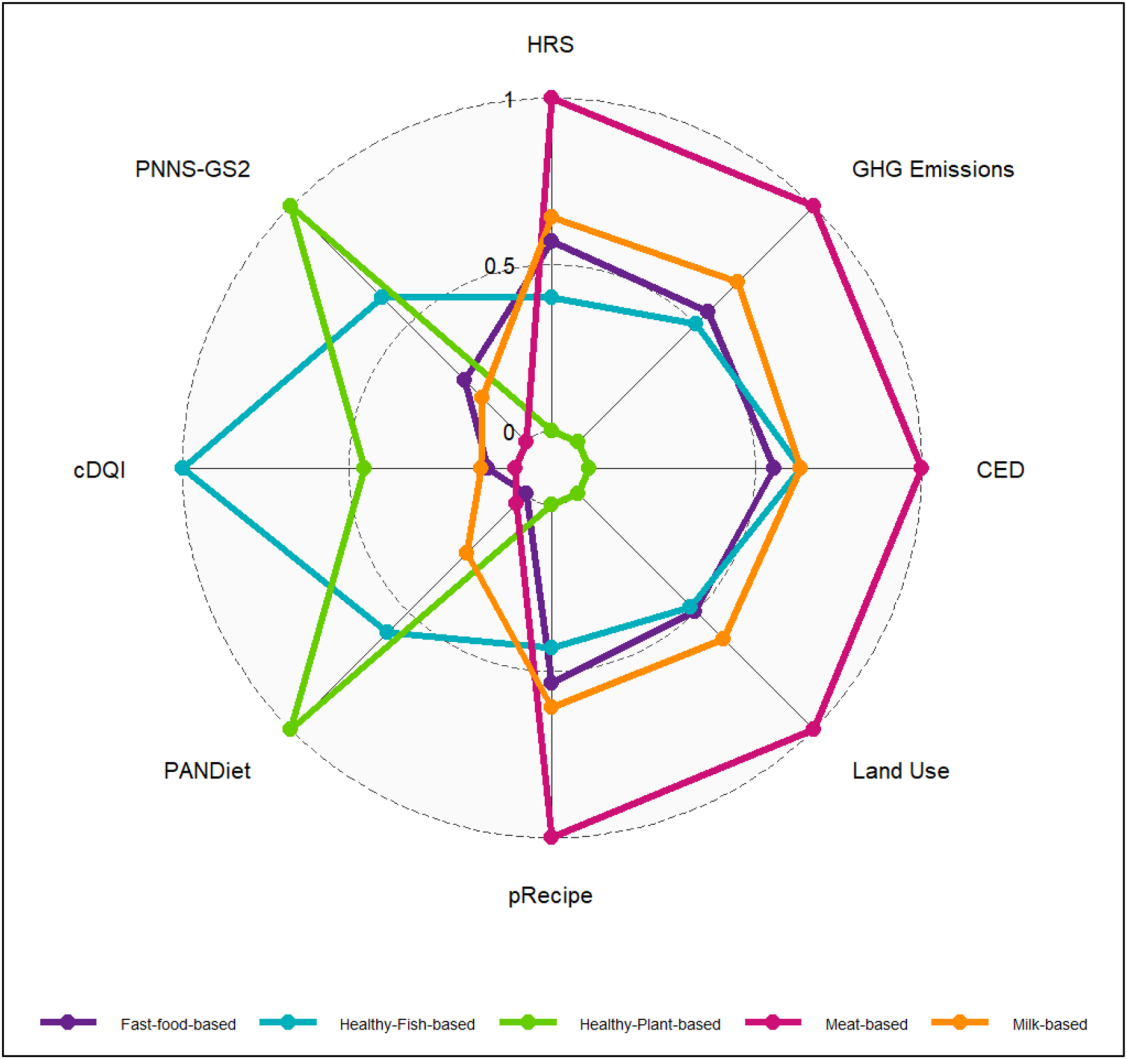
**Sustainability indicators across clusters**. The energy-adjusted means of indicators, computed using ANCOVA model, were rescaled to the same scale by equalizing the maximum value to 1 and the minimum value to 0 for each indicator. For the environmental indicators and the HRS, higher values denote higher impacts/risk. For nutritional quality indicators, higher values denote higher nutritional quality. *HRS* heath risk score, *GHG* greenhouse gas (kg CO2 eq/d), *CED* cumulative energy demand (MJ/d); Land Use (m^2^/d), *pRecipe* partial Recipe, *PANDiet* diet quality index based on the probability of adequate nutrient intake, *cDQI* comprehensive diet quality index, *PNNS-GS2* Programme National Nutrition Santé-Guidelines Score 2.

Results of the multicriteria analysis of clusters are presented in Table 2 and Fig. 2.

**Table 2:** Sustainability indicators across clusters.

Indicators	Whole sample	Milk-based	Meat-based	Fast-food-based	Healthy-fish-based	Healthy-plant-based	*p*
**Nutritional quality**							
PANDiet	64.97 (7.86)	65.17 (0.09)	63.01 (0.07)	62.65 (0.07)	68.53 (0.07)	72.68 (0.21)	< 0.0001
AS	78.86 (12.74)	78.43 (0.13)	78.11 (0.1)	75.73 (0.1)	83.79 (0.1)	77.64 (0.29)	< 0.0001
MS	51.08 (18.49)	51.9 (0.15)	47.9 (0.12)	49.57 (0.12)	53.27 (0.13)	67.72 (0.35)	< 0.0001
cDQI	51.48 (9.18)	49.57 (0.11)	48.56 (0.09)	49.33 (0.08)	58.21 (0.09)	52.97 (0.25)	< 0.0001
aDQI	15.86 (3.9)	16.84 (0.05)	14.59 (0.04)	15.73 (0.04)	17.05 (0.04)	13.16 (0.11)	< 0.0001
pDQI	35.62 (7.47)	32.72 (0.09)	33.96 (0.07)	33.6 (0.07)	41.16 (0.07)	39.81 (0.2)	< 0.0001
PNNS-GS2	2.51 (3.56)	1.96 (0.03)	1.01 (0.03)	2.34 (0.02)	4.15 (0.03)	6.13 (0.08)	< 0.0001
**Environmental impacts**							
GHG emissions (kg CO_2_ eq/d)	4.05 (2.48)	4.13 (0.02)	5.47 (0.02)	3.61 (0.01)	3.4 (0.02)	1.31 (0.05)	< 0.0001
Cumulative energy demand (MJ/d)	17.62 (7.56)	17.08 (0.06)	21.14 (0.05)	16.18 (0.04)	17.09 (0.05)	9.85 (0.14)	< 0.0001
Land use (m^2^/d)	10.6 (6.75)	10.57 (0.07)	14.24 (0.05)	9.33 (0.05)	9.15 (0.05)	4.52 (0.15)	< 0.0001
pRecipe	0.28 (0.16)	0.28 (0.00)	0.39 (0.00)	0.26 (0.00)	0.23 (0.00)	0.11 (0.00)	< 0.0001
Organic food share	0.29 (0.27)	0.21 (0.00)	0.21 (0.00)	0.26 (0.00)	0.41 (0.00)	0.67 (0.01)	< 0.0001
**Health risk**							
RR (%) whole grain	19.51 (12.35)	20.59 (0.16)	17.45 (0.13)	21.17 (0.12)	17.1 (0.14)	32.9 (0.37)	< 0.0001
RR (%) fruits	6.98 (7.59)	7.8 (0.10)	5.17 (0.08)	8.26 (0.08)	5.8 (0.08)	14.07 (0.23)	< 0.0001
RR (%) vegetables	2.19 (2.58)	2.87 (0.03)	1.54 (0.02)	2.77 (0.02)	1.69 (0.02)	2.4 (0.07)	< 0.0001
RR (%) nuts/seeds	3.82 (3.17)	3.84 (0.04)	2.95 (0.03)	3.88 (0.03)	4.1 (0.03)	7.56 (0.09)	< 0.0001
RR (%) legumes	10.24 (6.47)	10.29 (0.08)	7.63 (0.07)	10.63 (0.06)	12.21 (0.07)	12.14 (0.19)	< 0.0001
RR (%) milk	6.65 (5.86)	3.03 (0.06)	4.41 (0.05)	6.95 (0.05)	9.15 (0.05)	20.65 (0.14)	< 0.0001
RR (%) processed meat	16.87 (10.22)	16.58 (0.14)	18.26 (0.11)	17.73 (0.1)	16.47 (0.11)	3.75 (0.3)	< 0.0001
RR (%) red meat	32.97 (16.75)	34.08 (0.21)	41.97 (0.17)	27.67 (0.16)	32.89 (0.17)	5.19 (0.46)	< 0.0001
RR (%) sweetened beverages	0.73 (1.96)	0.86 (0.02)	0.58 (0.02)	0.88 (0.02)	0.53 (0.02)	1.29 (0.06)	< 0.0001
HRS	0.69 (0.29)	0.7 (0.00)	0.95 (0.00)	0.65 (0.00)	0.53 (0.00)	0.25 (0.00)	< 0.0001

Values are mean (SD) for the whole sample, and adjusted means of indicators on total energy intake (SEM) across clusters (ANCOVA model).

*P* values were calculated using ANCOVA.

*AS* adequation sub-score of PANDiet, *MS* moderation sub-score of PANDiet, *HRS* health risk score, *RR* relative risk.

### Nutritional quality

Table 2 shows that the healthy-plant-based had the highest PNNS-GS2 score (+ 144% compared to the whole sample), reflecting a better adherence to the PNNS guidelines and the highest PANDiet score (+ 12% compared to the whole sample) based on nutritional reference values, followed by the healthy-fish-based cluster. This latter cluster, though, showed the highest cDQI score (+ 15% compared to the whole sample), indicating the highest quality of both animal and plant foods consumed. Inversely, the meat-based cluster had the lowest nutritional scores for all computed indicators (− 61%, − 5% and − 8% respectively for the PNNS-GS2, the PANDiet, and the cDQI indicators).

### Health risk score

The health risk analysis (Table 2) showed that the structure of the healthy-plant-based cluster was the most beneficial compared to the other clusters (0.25 for the HRS score), followed by the healthy-fish-based cluster (0.53 for the HRS score). Yet, the health risk score associated with the meat-based cluster was the highest among the five clusters (0.95 for the HRS score). Furthermore, the analysis of the contribution of food groups to the health risk score shows that for all clusters, a low consumption of wholegrain products and legumes and a high consumption of red meat contribute the most to the value of the HRS.

### Environmental impacts

Table 2 shows that for all observed indicators, the healthy-plant-based cluster had the least impact on the environment (pReCIPe: − 62% compared to the whole sample), while the meat-based cluster showed the highest impact (pReCIPe: + 33% compared to the whole sample) among the five identified diets. The environmental impacts of healthy-fish-based and fast-food-based clusters were lower than the whole sample means for all indicators. The milk-based diet showed similar environmental impacts to the whole sample means.

### Organic food consumption across clusters

Table 2 shows that participants of the healthy-plant-based cluster had the highest share of organic consumption (+ 127% compared to the whole sample), followed by the participants of the healthy-fish-based cluster showing a 40% higher share than the average of the population. However, the participants of the meat-based and milk-based clusters showed the lowest share of organic food (− 30% compared to the whole sample) among the five identified clusters.

### Economic analysis

**Figure 3:**
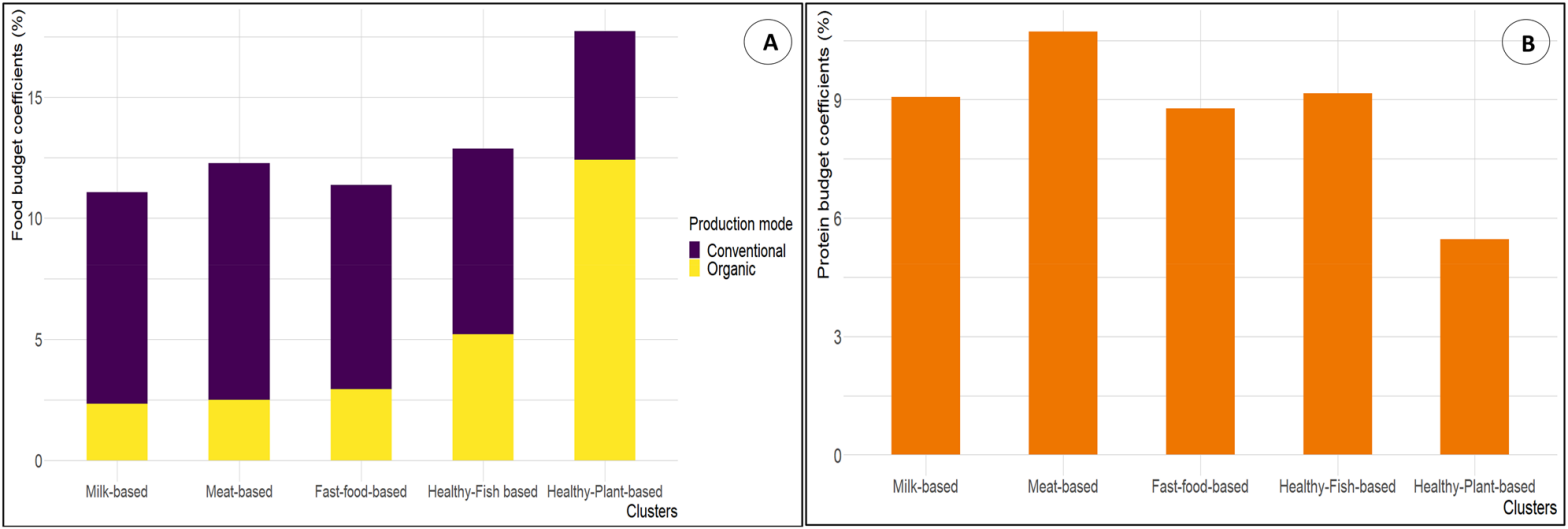
Food budget coefficients across clusters according to production mode (**A**); protein budget coefficients across clusters (**B**). Values are energy-adjusted means of budget coefficients computed using ANCOVA model. Panel A refers to food budget coefficients across clusters according to production mode (organic/conventional). Panel B refers to protein budget coefficients across clusters.

The healthy-plant-based cluster had the largest share of income allocated to food (+ 46% compared to the whole sample) (Fig. 3). On the opposite, consumers of milk-based and fast-food-based clusters spent the smallest share of their income for food (− 10% and − 6% compared to the whole sample), while the food budget coefficient of the meat-based cluster is similar to the mean of the population studied.

Comparison of clusters by food groups revealed that budget coefficients followed the same trend as food intake across the clusters. Furthermore, analysis of food expenditure structure by cluster showed that vegetables are the food group for which all clusters spent the largest share of their overall diet expenditure, with the exception of the meat-based cluster, who spent the most for meat.After vegetables, the milk-based cluster spent more for meat and non-alcoholic beverages, the fast-food-based cluster for dairy products, the healthy-fish-based cluster for fruit and seafood, and the healthy-plant-based cluster for fruits and soya-based foods. The detailed values are presented in Supplemental Table 3.

Analysis of the overall diet budget coefficients according to the production mode (Supplemental Table 4) showed that 70% of the food expenditure of the healthy-plant-based cluster was allocated to organic products, which contributes to the higher diet expenditure. Conversely, the meat-based cluster had the lowest share of the budget allocated to organic food (20%).

In terms of budget coefficients of protein intake (Fig. 3), meat-based cluster participants were those spending the most of their overall diet expenditure for their protein intake (+ 13% compared to the whole sample). The healthy-plant-based cluster exhibited the lowest protein expenditure (− 41% compared to the whole sample), followed by the fast-food-based and milk-based clusters (− 5% and − 3% respectively, compared to the whole sample). Analysis of protein expenditure structure by cluster showed that meat-based, milk-based and fast-food-based clusters spent the largest share of their overall diet expenditure on meat proteins. The healthy-fish-based cluster allocated the largest share of their overall diet expenditure to seafood proteins, while the healthy-plant-based cluster spent more on nuts proteins.

The detailed values are presented in Supplemental Table 5.

## Discussion

We extracted five clusters based according to food-group contribution to protein intake (all analyses are adjusted for energy intake). The healthy-plant-based cluster (3%) and the healthy-fish-based cluster (25%) were the most sustainable for the environmental, nutritional and health dimensions. Conversely, the meat-based cluster (26%) exhibited the highest environmental pressures, the lowest nutritional scores and a high health risk. Furthermore, based on an economic analysis, we observed that although the healthy-plant-based cluster had the highest food budget coefficient, its expenditure for protein intake was the lowest. Conversely, expenditure on protein intake was high for the meat-based cluster eaters. This study therefore argues that the protein sources of a diet are a good factor in the sustainability of diets.

### Nutritional quality and health risk across diets

The healthy-plant-based cluster exhibited the highest PANDiet score, and a better adherence to the PNNS guidelines. In fact, while the choice of protein sources in individuals’ diets often raises the question of protein adequacy (including protein and amino acid intakes)^16^, previous studies documented that balanced diets in accordance with public health goals and with low meat intake, provide an adequate intake for most nutrients^41^. Indeed, the amounts and quality of plant-based proteins are frequently underestimated or misunderstood^42^. Moreover, maintaining protein adequacy in spite of the decrease in the consumed quantity of animal protein could be explained by the great excess above the needs in terms of protein intake in Western countries^16^. But this issue is still being debated and for example, a Canadian cohort study stated that the transition to plant-based protein might be nutritionally challenging since animal protein contributes overwhelmingly to total protein intake, particularly for the elderly^43^.

The health risk analysis showed that the dietary structure of the healthy-plant-based cluster eaters is the most protective in terms of health benefits, as reflected using the HRS, while the health risk associated with the meat-based cluster is much higher. Similar results were found using another health risk estimator, the HiDiet score, aiming to evaluate the effect of diet on long-term morbidity and mortality^22^. Thus, plant-based protein consumption was proven to be associated with nutrient intakes and dietary profiles that are supportive of cardiometabolic health^44^. Moreover, the reduction in premature mortality associated with the adoption of balanced low-meat diets, was estimated at 19% for the flexitarian diet and 22% for the vegan diet^41^. Furthermore, the association between animal sourced food consumption and the risk of chronic diseases has been established by literature^45–47^. For instance, it has been demonstrated that a 5% substitution of animal protein with plant protein would reduce the risk of incidence of type 2 diabetes by 23%^48^. Indeed, red and processed meat were proved to be risk factors for type 2 diabetes, while soy and dairy products provide protection^49^.

### Environmental impacts across the diets

As the literature on the environmental impacts of protein consumption has not been sufficiently developed, we situated our results in relation to existing studies comparing predominantly animal-based diets to more plant-based diets. In our study, the healthy plant-based cluster had less environmental impact than the meat-based cluster, which exhibited the highest environmental pressures. This is in accordance with the available literature regarding the lowest impact of plant-based dietary patterns^8,22,50,51^. Moreover, it has been demonstrated, in a comprehensive review^52^, that the decrease of the environmental footprint is generally proportional to the extent to which animal-based food consumption is restricted^41^. Furthermore, we reported that belonging to the healthy-plant-based cluster was concomitant with a higher consumption of organic products, as shown before^53^, which could contribute to the lower environmental impacts, especially for energy demand. A previous study also based on the NutriNet-Santé cohort^54^, showed that organic food consumption could partly explain the inverse link between plant-based diet and some environmental impacts (GHG emissions and energy demand), specifying that this link is only established for individuals with diets rich in plant-based foods.

### Economic analysis

We showed that, at constant energy intake, the overall diet expenditure of the healthy-plant-based cluster was the highest among the five clusters, followed by the healthy-fish-based cluster, and to a lesser extent the meat-based cluster. In that regard, a previous study based on a representative sample of 1719 French adults (INCA2), showed that meeting with nutritional reference values systematically increased the cost of food^55^, which is consistent with our previous findings regarding the superiority of the nutritional quality of healthy-plant-based and healthy-fish-based clusters. Nevertheless, improving diet quality by optimization on nutritional constraints without increasing food expenditure, regardless of the initially observed cost, has been shown to be possible^55^. However, for food budgets below 3.85€/day, significant diet changes were needed. Furthermore, the high food budget coefficients associated with the healthy-plant-based and healthy-fish-based clusters might also be explained by the much higher share of organic food consumption of these two clusters. Similarly, another study also based on the NutriNet-Santé cohort^56^ demonstrated that high consumers of organic food displayed a high consumption of plant-based foods and a healthier diet. Thus, the monetary cost of their diet was higher (+ 26%) compared to that of low consumers due to the higher prices of organic products as shown by a decomposition model of the effects. This dual choice seems a best option by resulting in a markedly reduced exposure to pesticides from diet^56^.

Interestingly, the protein budget coefficient of the meat-based cluster was higher than that of other clusters, which could be explained by both the higher prices of protein foods characteristic of the diet adopted by this cluster’s participants (meat, poultry and processed meat) and their higher protein intake (+ 6% compared to the whole sample). Inversely, both the lower protein intake of the healthy-plant-based cluster eaters (− 27% compared to the whole sample) and the lower prices of their diet’s proteins sources (soya-based foods, legumes and nuts), might explain the lower protein budget coefficient. Indeed, it has been previously demonstrated in a meta-analysis assessing the nutritional status of meat-based diets compared to plant-based diets, that the protein intake of meat eaters was higher than that of people adopting a plant-based diet, although it was still within the recommended levels^57^.

### Multicriteria analysis of diets’ sustainability according to protein intake

The scarcity of studies on sustainability in its entirety is inherent to its multidimensionality, which makes it complex to conduct research in this sense. Indeed, while a multi-criteria analysis of protein profiles^22^ close to our study only addressed 2 of the 4 dimensions of diets sustainability (environment, nutrition and health) according to the FAO definition^21^, we also focused on the economic aspect since this dimension has rarely been accounted for in multi-criteria studies on sustainability. The economic analysis we carried out aimed to provide an initial overview of the aspect of economic affordability. However, cultural acceptability, which is a significant obstacle to achieving changes in dietary behaviour, haven't been sufficiently addressed neither in our work nor in that of Perraud et al.^22^. On the other hand, the multiplicity of aspects making up these dimensions prevent from covering them entirely. Indeed, although we assessed the environmental pressures and impacts on 3 aspects, the above-mentioned study^22^ evaluated other impacts by mobilizing more indicators, but without distinguishing organic and conventional foods as we did. The results of this study^22^ remain consistent with ours, showing that protein profiles associated with low meat consumption tend to be more sustainable on the two dimensions analysed, namely environment, nutrition and health. Moreover, due to the difference in dietary behaviours of the different populations considered in these two studies, discrepancies with our results are identified, notably in the identification of clusters (distinction between ruminant and monogastric meat in the protein profiles^22^, identification of more plant-based protein profiles in our work).

### Action levers for greater sustainability

Insofar as we concluded that the nature of protein intake is a discriminating factor in the sustainability of diets, it is relevant to consider this factor for the development of action plans for changing dietary patterns towards greater sustainability. As previously mentioned, meat consumption is associated with socio-cultural values, making transitions to plant-based diets more complex to manage in practice^58,59^. To this end, some suggestions have been developed in the literature. First, at the production stage, some environmental impacts could be reduced by integrating crops and livestock^60^ and promoting grazing systems. This could improve efficiency of animal feeding and nutrient cycling, besides crop rotation through temporary grasslands. Also, non-food biomass would serve as feed for animals, which provide organic fertilizer^60^. Then, at the consumption stage, an intrinsic change in diet at the food group level could be operated. As we demonstrated that beef, pork and poultry consumption are correlated for the meat-based cluster, replacing ruminant meat by poultry^13,61^ could contribute to reduce some environmental pressures, especially GHG emissions^13^ and to lower the health risk compared to red meat^13^. However, this raises the question of the individualized nature of dietary pattern, which conditions the feasibility of the effective transition from potential pathways to action plans for sustainable dietary changes. Acceptability is thus enhanced by considering personalized and targeted recommendations.

### Strengths and limitation

First of all, it is important to note that the participants in the NutriNet-Santé cohort are volunteers, who may have a greater interest in nutrition and health compared to the general population. As a result, this sample exhibits certain characteristics such as a higher proportion of women, older individuals, those with higher education and healthier dietary habits^62^. However, the large sample size allows access to a wide variety of dietary profiles and probably a higher representation of diets rich in plant-based foods. However, our sample is not representative of the French population and these results cannot be directly generalized. Secondly, the environmental data mobilized are limited to the production stage. However, this stage represents the major part of the environmental impacts of the food system. And, as mentioned above, the socio-cultural dimension associated with the choice of protein sources in diets was not considered in this study. However, our study is the first to provide a multi-criteria analysis of sustainability according to protein profiles, while including an economic analysis. It covers a large French population, with various dietary patterns, including plant-based diets. Moreover, our study considers production modes (organic, conventional), and the data on food expenditures are quite accurate by considering consumers' places of purchase.

## Conclusion

In conclusion, the nature of protein intake is a good discriminating factor of diets sustainability. The healthy-plant-based and healthy-fish-based clusters were the most sustainable, allowing to conciliate the trade-off between individual and environmental health. Conversely, the meat-based cluster exhibited the highest environmental pressures, the lowest nutritional scores and a higher health risk score. Additionally, although the healthy-plant-based cluster had the highest food budget coefficient, their expenditure on protein intake was the lowest. However, this same expenditure was high for the meat-based cluster, which is explained by both higher prices of the protein sources consumed and their higher protein intake. These results could be useful for the development of food transition strategies aimed at reducing animal protein consumption.

### Supplementary Information

Supplementary Information.

## Data Availability

Analytic code will be made available upon request pending to Dr Emmanuelle Kesse-Guyot (e.kesse@eren.smbh.univ-paris13.fr). Researchers from public institutions can submit a collaboration request including information on the institution and a brief description of the project to collaboration@etude-nutrinet-sante.fr. All requests will be reviewed by the steering committee of the NutriNet-Santé study. If the collaboration is accepted, a data access agreement will be necessary and appropriate authorizations from the competent administrative authorities may be needed. In accordance with existing regulations, no personal data will be accessible.

## Abbreviations

IPCC: Intergovernmental Panel on Climate Change
GBD: Global Burden of Diseases
INSEE: National Institute of Statistics and Economic Studies
Org-FFQ: Organic Food Frequency Questionnaire
GHG: Greenhouse gas
CED: Cumulative energy demand
pReCiPe: Partial ReCiPe
PANDiet: Diet Quality Index based on the Probability of Adequate Nutrient Intake
PNNS: Programme National Nutrition Santé
PNNS-GS2: Programme National Nutrition Santé-Guidelines Score 2
cDQI: Comprehensive Diet Quality Index
AS: Adequation sub-score of PANDiet
MS: Moderation sub-score of PANDiet
TMREL: Theorical Minimum-Risk Exposure Level
HRS: Health risk score
SD: Standard deviation
SEM: Standard error of the mean
AHC: Ascending Hierarchical Classification
PCA: Principal Component Analysis
SFF: Sweetened and fatty foods
