# Supplementary material for: The nature of protein intake as a discriminating factor of diet sustainability: a multi-criteria approach
Source: Sci Rep. 2023 Oct 19;13:17850. doi: 10.1038/s41598-023-44872-3 (PMC10587119; doi:10.1038/s41598-023-44872-3)
Supplement: Supplementary file 1 — Supplementary Information. [file 41598_2023_44872_MOESM1_ESM.docx]

# Online Supplemental material

## Supplemental Method 1: Computation of the environmental indicators

The analysis scope considered by the DIALECTE tool (1) is limited to the agricultural production stage. The production of inputs and the supply of energy are included in this scope, while the processing, packaging, transport, storage and recycling phases are excluded. This tool contains information from 2,086 farms with different production systems, 46% of which are certified organic.

Thereby, in the NutriNet-Santé study (2,3), these indicators were calculated by multiplying the individual food consumption of each product by the corresponding values of the environmental indicators and conversion factors. The conversion factors allow for the conversion of agricultural products into food products in order to be able to estimate the environmental impacts associated with food as consumed. Economic allocations by co-product were applied, followed by mass, cooking and edibility coefficient assignments. Then by summing all the food consumed, considering the production mode, the environmental impact of the overall diet is obtained. Economic allocations by co-product were applied, followed by mass, cooking and edibility coefficient assignments. Hence, indicators’ values for the ingredient as consumed were obtained.

The pReCiPe score, a synthetic impact indicator, has been calculated (4). It corresponds to a partial ReCiPe indicator. To balance conflicting environmental indicators, the ReCiPe method considers both midpoint and endpoint measures. Developed in the Netherlands, this LCA method aligns the indicators to provide a comprehensive view (5). It focuses on 18 indicators, three of which are oriented towards final impacts, including resource availability, human health and ecosystem diversity. In practice, some authors have found that the environmental impact of food products and diets can be assessed by measuring greenhouse gas emissions, primary energy consumption, and land occupation. These factors make up about 90% of the total environmental dimension of the ReCiPe model. To calculate the environmental impact of a food product or diet, one can use the partial ReCiPe score (pReCiPe), with normalization and weighting factors (4).

## Supplemental Method 2: Computation of dietary indexes

The nutritional quality of individual diets was assessed using the following scores:

The ***PANDiet*** (Diet Quality Index based on the Probability of Adequate Nutrient Intake) score describes the nutritional quality of a diet (6,7). Its value ranges from 0 to 100, and includes 28 nutrients, aggregating the means of two sub-scores, namely adequacy score (***AS***) and moderation score (***MS***). The AS is the average probability that nutrient intakes meet the reference values for the following nutrients: total fat, fiber, protein, vitamins (A, B1, B2, B3, B5, B6, B9, B12, C, D and E), bioavailable iron, copper, iodine, calcium, magnesium, manganese, phosphorus, potassium, bioavailable zinc, selenium, n-3 and n-6 fatty acids, docosahexaenoic acid and eicosapentaenoic acid. The MS is the average probability that nutrient intakes will not exceed the reference value, determined by the nature of the nutrient, for the following nutrients: vitamins B3, B6, B9, D and E, retinol calcium, magnesium, copper, selenium, iodine and zinc.

The ***PNNS-GS2*** (National Nutrition Health Guidelines Score) measures the adherence of individuals to the French dietary recommendations established by the HCSP (High Council of Public Health) in 2017 (8) and launched in 2019. It includes 13 components referring to two food categories. Healthy foods whose consumption increases the score, including fruits and vegetables, wholegrain foods, legumes, milk and dairy products, nuts, fish and seafood, and added fats that are oils rich in a-linolenic acid; and foods whose consumption should be limited, as it leads to negative points, namely sugary foods, red meat, processed meat, sweetened beverages, alcoholic beverages and salt. The reference portions were determined according to the portions usually consumed in France. An expertise agreed to set the scores and thresholds between 0 and 2 to reflect compliance with the recommendations for healthy foods, and between 0 and -2 for foods to be limited. In addition, a dimension of organic food consumption was considered in the score for plant foods, notably breads and cereals, fruits and vegetables and legumes, by attributing malus and bonus. Weighting was applied between components according to the evidence established for the relationship with health, based on an expert panel (9). In the case where energy intake exceeds energy expenditure by more than 5%, a penalty is deducted from the score (PNNS-GS penalized). Thus, the final score can range from -∞ to 14.25.

The ***cDQI*** (Comprehensive Diet Quality Index) aims to assess the quality of diets by evaluating their plant and animal components (10).

The ***pDQI*** (Plant-based Diet Quality Index) focuses on the quality of plant-based foods, considering two groups of foods, namely healthy foods (wholegrain products, fruits, vegetables except potatoes, nuts, seeds and legumes, vegetable oils, coffee and tea) and foods to be limited (refined grains, fruit juices, potatoes, sweetened beverages and foods).

The ***aDQI*** (Animal-based Diet Quality Index), describes the quality of foods of animal origin. It includes two groups of foods, healthy (fish and seafood, dairy products and poultry) and those to be limited (red meat, processed meat and eggs). For each food, a score ranging from 0 to 5 is assigned depending on whether the consumption of the considered food corresponds to the reference consumption. Thus, the final scores vary between 0 and 55 for pDQI, and from 0 to 30 for aDQI. The final cDQI score is obtained by adding the two pDQI and aDQI scores, and varies between 0 and 85.

## Supplemental Method 3: Computation of the Health Risk Score

The Global Burden Of Disease (GBD) study aims to describe the morbidity and mortality of major diseases and health risk factors worldwide (11). Conducted by the IHME (Institute for Health Metrics and Evaluation), it is the most comprehensive global observational epidemiological study to understand the health challenges facing people worldwide in the 21st century. The TMREL (Theoretical Minimum-Risk Exposure Level), defined in the GBD study, refers to the level of exposure that minimizes the risk of death involved by all the causes associated with a single risk factor (in this case over- or under-consumption of a food or nutrient). The aim is to estimate the optimal intake of each factor using an objective approach, rather than the conventional subjective approach based on expert opinion. To do this, the GBD study reviewed numerous studies to assess the relationship between each risk factor and disease indicator, then the intake level associated with the lowest risk of mortality for that indicator was calculated to obtain a disease-specific optimal intake level. The TMREL equals the average of these optimal values weighted by the number of deaths caused by each disease worldwide. In other words, the TMREL is the optimal level of a food consumption that minimizes the risk of death from an over- or an under-consumption of that food.

Thereby, the diet Health Risk Score (HRS) (12) constructed from the distance to the TMREL, used in this study, corresponds to the risk associated with the overall diet, and it is calculated with the formula:

$$\text{HRS}=\sum_{\text{i=1}}^{\text{3}} \left( \frac{\text{Cons }\left( \text{i} \right)}{\text{Max(}\text{i}\text{)}}\times\frac{\text{DALYs}\left( \text{i} \right)}{\text{DALYs}\left( \text{all} \right)} \right) + \sum_{\text{j=1}}^{\text{6}} \text{ }\left( \text{max}\left[ \frac{\text{TMREL(j) - Cons }\left( \text{j} \right)\text{ }}{\text{TMREL(j)}}\text{; 0} \right]\text{×}\frac{\text{DALYs}\left( \text{j} \right)}{\text{DALYs}\left( \text{all} \right)} \right)$$

*Where:*

- **i:** food groups to be restricted (red meat, processed meat et sweetened beverages);
- **j:** healthy food groups to be promoted (wholegrain foods, fruits, vegetables, legumes, nuts and grains, milk);
- **Max(i)**: upper limit of consumption of the food group i (g/d);
- **TMREL(j)**: TMREL value for the food group j (g/d);
- **DALYs(i)**, **DALYs(j)**: disability-adjusted life years associated with over-/under-consumptions of the food groups i and j respectively (in years);
- **DALYs(all)**: total of all DALYs(i) and DALYs(j).

The TMREL and corresponding DALYs values are presented below.

**Table 3: Theoretical minimum-risk exposure level (TMREL) and disability-adjusted life-years (DALYs) values used in the optimization model in males and females.**

|  |  | **TMREL^1^**  **(g/d)** | |  |  |  | **DALYs^2^**  **(y)** | |
| --- | --- | --- | --- | --- | --- | --- | --- | --- |
|  |  | **Males** | **Females** |  |  |  | **Males** | **Females** |
| **Unhealthy**  **foods** | **Red meat** | 0 | 0 |  |  |  | 28 562 | 20 824 |
|  | **Processed meat** | 0 | 0 |  |  |  | 14 346 | 6 288 |
|  | **Sweetened beverages** | 0 | 0 |  |  |  | 4 105 | 1 791 |
| **Healthy**  **foods** | **Whole grains** | 170 | 137 |  |  |  | 31 405 | 10 987 |
|  | **Fruit** | 367 | 297 |  |  |  | 20 130 | 9 512 |
|  | **Legumes** | 107 | 87 |  |  |  | 17 103 | 3 386 |
|  | **Vegetables** | 339 | 274 |  |  |  | 9 342 | 3 090 |
|  | **Nuts and seeds** | 16 | 13 |  |  |  | 6 531 | 1 355 |
|  | **Milk** | 486 | 393 |  |  |  | 3 521 | 2 727 |
|  | **Total** |  |  |  |  |  | 135 045 | 59 961 |

^1^According to the most recent (2019) estimates from the GBD, the TMREL values are of 0 g/d for red meat, processed meat and sweetened beverages, and of 150, 325, 95, 300, 14.5 and 430 g/d respectively for whole cereal products, fruits, legumes, vegetables, nuts and seeds, and milk. As these TMREL values are global estimates corresponding to a mean energy intake of 2,300 kcal (13), we used gender-specific values adapted to the particular energy intake of men and women in our French population (centered around 2600 kcal and 2100 kcal in men and women, respectively).

^2^We used the most recent (2019) French gender-specific DALYs values associated with excessive/insufficient consumptions of unhealthy/healthy foods, available from the Global Health Data Exchange website (<http://ghdx.healthdata.org/gbd-results-tool>).

The computation is done in 3 steps. First, for each food group, the distance to the TMREL is calculated, and refers to the adequacy of the quantity consumed to the reference level of the TMREL. For healthy food groups, a high consumption (above the TMREL) corresponds to a score of 0, while a consumption below the TMREL results in a score strictly greater than 0 and less than or equal to 1. Consequently, the higher the score, the worse the health impact. For foods to be limited, consumption below the reference portion results in a score between 0 and 1, while overconsumption is associated with a score strictly greater than 1. Thus, values obtained range between 0 and 1.

Then, the calculated distance is weighted by the ratio between food specific DALYs (Disability-Adjusted Life Years) to total DALYs of all foods. This reflects the risk of death related to a suboptimal consumption of a food, weighted by the years of life lost due to this food specific consumption (sum of years lived with disability and years lost due to early death). The interpretation is similar to the first step for healthy and unhealthy food groups. The value of the HRS estimator is obtained by summing the weighted distances.

Finally, in order to obtain the contribution (in %) to the overall risk of death (risk weighted by DALYs) represented by the suboptimal consumption of each food group, the ratio (in %) between the relative risk per food (result of the 2nd step) and the overall relative risk associated with the overall diet (HR) is calculated.

## Supplemental Method 4: Economic data

Participants' income was collected as part of the socio-economic status questionnaire, where each participant declared the income class corresponding to their monthly income. Income per consumption unit (C.U) was calculated using the household income class and the household composition (number of persons and age) according to the INSEE scale (14). One household unit was assigned to the first adult in the household, 0.5 to other individuals aged ≥14 years, and 0.3 to children aged <14 years. Household income was classified into 5 groups (<1200 €/month; 1200-1800 €/month; 1800-2700 €/month; >2700 €/month; and refusal to declare).

In the NutriNet-Santé study, the monetary cost of the diet (€/d) was calculated for each participant by multiplying the quantities of food consumed (g/d) by the corresponding prices (€/g), considering edible part and culinary preparations. The prices of the products were determined according to the individuals' places of purchase, organic and conventional. The data on the places of purchase (supermarkets, markets, AMAP (associations for the maintenance of a peasant agriculture), specialized organic stores, craftsmen, etc.) were collected by a complementary questionnaire on the NutriNet-Santé platform. Then, prices for each of the 264 frequency questionnaire foods and for each place of purchase were calculated on the basis of average prices derived from Kantar Worldpanel 2012 (15), which is a purchase database that includes home-scan data from 20,000 French households. In addition, the prices of products purchased in short circuits were collected by the association Bioconsom'acteurs (2).

## Supplemental Method 5: Construction of the protein-source-typology

In order to classify individuals based on their primary sources of protein, hierarchical agglomerative clustering was employed, starting from the factors identified by PCA. The hierarchical clustering was conducted with data preprocessing using the K-means algorithm, which was iterated 100 times. Given that our study relies on a substantial database, the combined use of both k-means and hierarchical clustering methods allows to stabilize the solution.

First, the K-means algorithm, an iterative segmentation process, is used. It begins by randomly initializing "k" cluster centers and allocating the closest individuals based on distance. The centers are recalculated at each iteration until the segmentation is stabilized.

Hence, in the context of our analysis, where CAH is performed on the results of k-means clustering, the process begins by calculating the distances between the classes of individuals defined by k-means clustering.

Then, a first cluster containing the two classes that minimize the dissimilarity criterion (Euclidean distance between the centers of the classes) is created. The distance between this cluster and the other n-2 classes is calculated. Subsequently, the two classes whose combination minimizes the dissimilarity criterion are merged. This process continues until all the classes are merged.

The clustering was performed using the “HCPC” function of the “FactoMineR” package in “Rstudio”. The “kk” argument of this function was used and corresponds to the number of clusters used in a K-means preprocessing before the hierarchical clustering (16)

## Supplemental Table 1: Participant characteristics across clusters, n = 29 210, NutriNet-Santé Study^1,2^

|  | **Whole Sample** | **Milk-based** | **Meat-based** | **Fast-food-based** | **Healthy-fish-based** | **Healthy-plant-based** | **P** |
| --- | --- | --- | --- | --- | --- | --- | --- |
|  | n = 29,210 | n = 4,966 (17%) | n = 7,569 (26%) | n = 8,469 (29%) | n = 7,189 (25%) | n = 1,017 (3%) |  |
| **Sex.** |  |  |  |  |  |  | < 0.0001 |
| Men | 25.26 | 26.12 | 31.56 | 22.80 | 21.17 | 23.50 |  |
| Women | 74.74 | 73.88 | 68.44 | 77.20 | 78.83 | 76.50 |  |
| **Age** | 53.55 (13.97) | 51.95 (15.14) | 54.43 (13.29) | 51.17 (14.18) | 57.75 (11.99) | 44.67 (14.51) | < 0.0001 |
| **Occupational position** |  |  |  |  |  |  | < 0.0001 |
| Self-employed/farmer | 1.75 | 1.19 | 1.89 | 1.66 | 1.74 | 4.13 |  |
| Managerial staff/intellectual profession | 21.01 | 20.96 | 20.29 | 23.99 | 17.75 | 24.88 |  |
| Unemployed | 9.00 | 8.22 | 8.55 | 8.64 | 9.51 | 15.54 |  |
| Employee. manual worker | 14.31 | 16.03 | 15.44 | 14.94 | 10.63 | 18.29 |  |
| Students | 1.93 | 2.80 | 1.33 | 2.35 | 0.75 | 7.08 |  |
| Intermediate professions | 14.70 | 15.83 | 13.37 | 17.19 | 12.42 | 14.36 |  |
| Retired | 37.30 | 34.98 | 39.12 | 31.22 | 47.20 | 15.73 |  |
| **Monthly income per household unit** |  |  |  |  |  |  | < 0.0001 |
| NA | 6.05 | 5.82 | 5.64 | 5.76 | 6.68 | 8.06 |  |
| < 1200€/C.U. | 11.56 | 12.18 | 11.07 | 11.61 | 9.70 | 25.07 |  |
| 1200 - 1800€/ C.U. | 23.11 | 23.80 | 24.34 | 23.45 | 20.87 | 23.60 |  |
| 1800 - 2700€/ C.U. | 27.50 | 28.21 | 26.69 | 27.29 | 28.91 | 21.83 |  |
| >2700€/ C.U. | 31.78 | 29.98 | 32.26 | 31.89 | 33.86 | 21.44 |  |
| **Place of residence** |  |  |  |  |  |  | < 0.0001 |
| Rural community | 22.59 | 22.88 | 24.01 | 21.44 | 22.52 | 20.65 |  |
| Urban unit (<20,000 inhabitants) | 15.33 | 15.00 | 16.66 | 14.76 | 15.19 | 12.78 |  |
| Urban unit (20,000 to 200,000 inhabitants) | 18.19 | 18.24 | 17.78 | 18.43 | 18.58 | 16.03 |  |
| Urban unit (>200,000 inhabitants) | 43.90 | 43.88 | 41.55 | 45.37 | 43.71 | 50.54 |  |
| **Smoking habits** |  |  |  |  |  |  | < 0.0001 |
| Never smoker | 48.78 | 55.84 | 45.18 | 49.88 | 45.95 | 52.11 |  |
| Former smoker | 40.47 | 35.12 | 42.13 | 38.25 | 45.57 | 36.68 |  |
| Current smoker | 10.75 | 9.04 | 12.68 | 11.88 | 8.49 | 11.21 |  |
| **Physical activity** |  |  |  |  |  |  | < 0.0001 |
| Low | 19.20 | 20.48 | 21.80 | 20.72 | 14.30 | 15.63 |  |
| Moderate | 36.38 | 35.90 | 34.81 | 37.93 | 35.97 | 40.41 |  |
| High | 33.64 | 32.46 | 32.32 | 30.17 | 39.42 | 37.27 |  |
| Missing data | 10.78 | 11.16 | 11.07 | 11.18 | 10.31 | 6.69 |  |
| **BMI** |  |  |  |  |  |  | < 0.0001 |
| Underweight | 4.68 | 4.37 | 2.63 | 5.16 | 5.17 | 13.86 |  |
| Healthy | 59.86 | 59.67 | 52.01 | 62.95 | 62.80 | 72.57 |  |
| Overweight | 25.42 | 25.63 | 31.51 | 23.14 | 23.69 | 10.13 |  |
| Obese | 10.05 | 10.33 | 13.85 | 8.75 | 8.33 | 3.44 |  |
| **BMI** | 24.20 (4.63) | 24.28 (4.51) | 25.19 (4.74) | 23.87 (4.64) | 23.84 (4.47) | 21.79 (3.53) | < 0.0001 |

Abbreviations: BMI, body mass index; C.U., consumption unit

^1^Values are mean (SD) or % as appropriate.

^2^P values were calculated using ANOVA or Chi² test

## Supplemental Table 2: Nutrient intakes across clusters^1,2,3^

| **Nutrients** | **Whole Sample** | **Milk-based** | **Meat-based** | **Fast-food-based** | **Healthy-fish-based** | **Healthy-plant-based** | **p** |
| --- | --- | --- | --- | --- | --- | --- | --- |
| **Total energy intake (kcal/d)** | 2001 (629) | 2019 (600) | 2041 (654) | 1983 (642) | 1968 (602) | 1998 (635) | < 0.0001 |
| **Total protein intake (g/d)** | 91.11 (33.98) | 95.8 (0.25) | 98.77 (0.20) | 85.9 (0.19) | 89.25 (0.21) | 66.82 (0.56) | < 0.0001 |
| **Plant-based protein (g/d)** | 28.64 (12.41) | 25.34 (0.11) | 24.75 (0.09) | 27.14 (0.08) | 33.40 (0.09) | 52.60 (0.24) | < 0.0001 |
| **Probability of adequate protein intake** | 0.95 (0.18) | 0.97 (0.13) | 0.96 (0.15) | 0.93 (0.21) | 0.94 (0.18) | 0.83 (0.32) | < 0.0001 |
| **Carbohydrates (% of total energy intake)** | 38.48 (7.63) | 40.33 (0.10) | 35.75 (0.08) | 39.31 (0.07) | 38.06 (0.08) | 45.84 (0.22) | < 0.0001 |
| **Lipids (% of total energy intake)** | 39.98 (7.00) | 37.70 (0.09) | 40.45 (0.07) | 40.48 (0.07) | 40.64 (0.08) | 38.87 (0.21) | < 0.0001 |
| **Polyunsaturated fat (g/d)** | 6.64 (2.48) | 5.64 (0.03) | 6.31 (0.02) | 6.09 (0.02) | 7.81 (0.02) | 10.07 (0.07) | < 0.0001 |
| **Monounsaturated fatty acids (g/d)** | 15.9 (3.98) | 14.50 (0.05) | 16.11 (0.04) | 15.68 (0.04) | 16.79 (0.04) | 16.63 (0.12) | < 0.0001 |
| **Saturated fatty acids (g/d)** | 14.47 (3.54) | 14.77 (0.04) | 14.98 (0.03) | 15.72 (0.03) | 12.98 (0.03) | 9.33 (0.10) | < 0.0001 |
| **n-3 fatty acids** | 0.95 (0.55) | 0.76 (0.00) | 0.85 (0.00) | 0.81 (0.00) | 1.29 (0.00) | 1.31 (0.01) | < 0.0001 |
| **Alpha linolenic acids** | 0.69 (0.49) | 0.54 (0.00) | 0.59 (0.00) | 0.6 (0.00) | 0.92 (0.00) | 1.22 (0.01) | < 0.0001 |
| **Eicosapentaenoic acid** | 0.08 (0.08) | 0.07 (0.00) | 0.08 (0.00) | 0.07 (0.00) | 0.12 (0.00) | 0.02 (0.00) | < 0.0001 |
| **Docosahexaenoic acid** | 0.11 (0.09) | 0.09 (0.00) | 0.11 (0.00) | 0.09 (0.00) | 0.16 (0.00) | 0.03 (0.00) | < 0.0001 |
| **n-6 fatty acids** | 5.37 (2.19) | 4.58 (0.02) | 5.13 (0.02) | 4.96 (0.02) | 6.22 (0.02) | 8.5 (0.06) | < 0.0001 |
| **Linoleic acid** | 5.31 (2.2) | 4.52 (0.02) | 5.05 (0.02) | 4.9 (0.02) | 6.16 (0.02) | 8.48 (0.06) | < 0.0001 |
| **Arachidonic acid** | 0.06 (0.02) | 0.06 (0.00 | 0.08 (0.00) | 0.05 (0.00) | 0.06 (0.00) | 0.01 (0.00) | < 0.0001 |
| **Fiber (g/d)** | 23.35 (8.66) | 20.71 (0.10) | 20.3 (0.08) | 21.12 (0.08) | 28.87 (0.08) | 38.47 (0.23) | < 0.0001 |
| **Vitamin A (µg eq retinol/d)** | 934.95 (741.02) | 893.18 (10.48) | 996.68 (8.49) | 870.42 (8.02) | 984.79 (8.71) | 864.71 (23.16) | < 0.0001 |
| **Vitamin B1 (mg/d)** | 1.44 (0.43) | 1.5 (0.00) | 1.41 (0.00) | 1.31 (0.00) | 1.54 (0.00) | 1.85 (0.01) | < 0.0001 |
| **Vitamin B2 (mg/d)** | 2.26 (0.59) | 2.66 (0.00) | 2.19 (0.00) | 2.15 (0.00) | 2.22 (0.00) | 2.03 (0.01) | < 0.0001 |
| **Vitamin B-3/total PP (mg/d)** | 24.67 (7.10) | 22.75 (0.09) | 27.38 (0.07) | 22.32 (0.07) | 26.54 (0.07) | 20.35 (0.20) | < 0.0001 |
| **Vitamin B5 (mg/d)** | 6.48 (1.38) | 7.25 (0.01) | 6.47 (0.01) | 5.99 (0.01) | 6.6 (0.01) | 6.05 (0.04) | < 0.0001 |
| **Vitamin B6 (mg/d)** | 2.05 (0.50) | 1.95 (0.00) | 2.09 (0.00) | 1.83 (0.00) | 2.27 (0.00) | 2.45 (0.01) | < 0.0001 |
| **Vitamin B9/Folic acid (µg/d)** | 419.47 (142.81) | 381.13 (1.84) | 384.19 (1.49) | 390.87 (1.41) | 487.42 (1.53) | 626.95 (4.08) | < 0.0001 |
| **Vitamin B12 (µg/d)** | 6.5 (5.08) | 6.8 (0.07) | 7.57 (0.05) | 5.53 (0.05) | 6.87 (0.05) | 2.53 (0.15) | < 0.0001 |
| **Vitamin C (mg/d)** | 152.49 (82.57) | 142.58 (1.14) | 138.14 (0.92) | 147.85 (0.87) | 171.19 (0.95) | 214.01 (2.53) | < 0.0001 |
| **Vitamin D (µg/d)** | 3.01 (1.77) | 2.67 (0.02) | 3 (0.01) | 2.77 (0.01) | 3.75 (0.02) | 1.46 (0.05) | < 0.0001 |
| **Vitamin E (mg/d)** | 14.57 (5.45) | 13.12 (0.07) | 13.56 (0.05) | 13.46 (0.05) | 16.98 (0.05) | 21.41 (0.15) | < 0.0001 |
| **Vitamin K (mg/d)** | 241.74 (174.94) | 198.39 (2.37) | 226.61 (1.92) | 205.2 (1.81) | 309.23 (1.97) | 393.31 (5.24) | < 0.0001 |
| **Calcium (mg/d)** | 1115.11 (329.86) | 1386.99 (4.22) | 971.28 (3.42) | 1150.46 (3.23) | 1066.34 (3.50) | 908.37 (9.32) | < 0.0001 |
| **Iron (mg/d)** | 15.48 (3.75) | 14.92 (0.04) | 14.70 (0.03) | 14.05 (0.03) | 17.7 (0.03) | 20.22 (0.10) | < 0.0001 |
| **Iodine (µg/d)** | 255.81 (407.34) | 236 (5.64) | 198.76 (4.57) | 212.58 (4.32) | 327.38 (4.69) | 631.33 (12.47) | < 0.0001 |
| **Magnesium (mg/d)** | 480.89 (138.95) | 455.39 (1.85) | 449.11 (1.50) | 452.11 (1.41) | 548.44 (1.54) | 604.05 (4.09) | < 0.0001 |
| **Phosphorus (mg/d)** | 1478.4 (278.16) | 1615.73 (3.81) | 1448.89 (3.08) | 1415.39 (2.91) | 1504.93 (3.16) | 1364.61 (8.42) | < 0.0001 |
| **Potassium (mg/d)** | 3808.98 (865.73) | 3954.63 (11.73) | 3712.8 (9.51) | 3492.34 (8.98) | 4120.59 (9.75) | 4247.52 (25.93) | < 0.0001 |
| **Sodium (mg/d)** | 2502.37 (546.78) | 2503.17 (7.47) | 2599.26 (6.06) | 2580.54 (5.72) | 2398.83 (6.21) | 1858.26 (16.52) | < 0.0001 |
| **Copper (mg/d)** | 2.09 (0.88) | 1.82 (0.01) | 1.99 (0.00) | 1.91 (0.00) | 2.46 (0.00) | 2.88 (0.02) | < 0.0001 |
| **Zinc (mg/d)** | 12.75 (2.61) | 13.2 (0.03) | 13.5 (0.02) | 12.00 (0.02) | 12.77 (0.02) | 11.23 (0.07) | < 0.0001 |
| **Manganese (mg/d)** | 5.17 (2.30) | 4.49 (0.02) | 4.16 (0.02) | 4.75 (0.02) | 6.74 (0.02) | 8.43 (0.06) | < 0.0001 |
| **Selenium (µg/d)** | 81.28 (21.25) | 77.19 (0.29) | 83.54 (0.23) | 75.62 (0.22) | 89.49 (0.24) | 73.46 (0.64) | < 0.0001 |

^1^Values are mean (SD) for the whole sample, and energy-adjusted means of nutrients intake (SEM) across clusters (ANCOVA model).

^2^P values were calculated using ANCOVA.

^3^ Prevalence of adequate protein intake calculated according the weight of the individuals: values are mean (SD) for the whole sample and the clusters.

## Supplemental Table 3: Food budget coefficients (%) across clusters^1,2^

| **Food groups (g/d)** | **Whole sample** | **Milk-based** | **Meat-based** | **Fast-food-based** | **Healthy-fish-based** | **Healthy-plant-based** | **p** |
| --- | --- | --- | --- | --- | --- | --- | --- |
| **Whole diet** | 12.13 (8.52) | 11.07 (0.11) | 12.27 (0.08) | 11.36 (0.08) | 12.88 (0.09) | 17.74 (0.24) | < 0.0001 |
| **Vegetables** | 15.45 (9.69) | 13.65 (0.13) | 13.62 (0.10) | 13.9 (0.10) | 19.15 (0.11) | 25.22 (0.30) | < 0.0001 |
| **Nuts** | 1.8 (2.96) | 1.13 (0.03) | 0.9 (0.03) | 1.34 (0.03) | 3.12 (0.03) | 6.5 (0.08) | < 0.0001 |
| **Dairy** | 10.91 (7.31) | 10.34 (0.09) | 9.6 (0.08) | 14.5 (0.07) | 9.47 (0.08) | 3.49 (0.22) | < 0.0001 |
| **Poultry** | 3.24 (3.09) | 3.22 (0.04) | 4.74 (0.03) | 2.61 (0.03) | 2.81 (0.03) | 0.37 (0.09) | < 0.0001 |
| **Meat** | 10.55 (8.17) | 11.28 (0.10) | 16.73 (0.08) | 8.2 (0.07) | 7.65 (0.08) | 0.78 (0.22) | < 0.0001 |
| **Processed meat** | 3.35 (3.05) | 3.41 (0.04) | 4.38 (0.03) | 3.45 (0.03) | 2.51 (0.03) | 0.36 (0.09) | < 0.0001 |
| **Potatoes** | 0.69 (0.78) | 0.67 (0.01) | 0.87 (0.00) | 0.62 (0.00) | 0.57 (0.00) | 0.67 (0.02) | < 0.0001 |
| **Cereals** | 5.76 (4.09) | 6.12 (0.05) | 5.86 (0.04) | 6.7 (0.04) | 4.08 (0.04) | 7.13 (0.12) | < 0.0001 |
| **Beverages** | 10.85 (6.99) | 12.72 (0.09) | 10.62 (0.07) | 10.96 (0.07) | 9.96 (0.08) | 8.6 (0.21) | < 0.0001 |
| **Alcohol** | 5.48 (6.94) | 4.76 (0.10) | 6.64 (0.08) | 5.34 (0.07) | 5.24 (0.08) | 3.04 (0.22) | < 0.0001 |
| **Soya-based food** | 1.54 (4.4) | 0.55 (0.05) | 0.48 (0.04) | 0.91 (0.04) | 2.44 (0.04) | 13.52 (0.12) | < 0.0001 |
| **Fat** | 0.18 (0.25) | 0.15 (0.00) | 0.15 (0.00) | 0.24 (0.00) | 0.16 (0.00) | 0.25 (0.00) | < 0.0001 |
| **SFF** | 7.15 (4.91) | 7.72 (0.06) | 6.44 (0.05) | 8.69 (0.05) | 5.81 (0.05) | 6.11 (0.15) | < 0.0001 |
| **Fruits** | 11.92 (10.13) | 10.57 (0.14) | 10.15 (0.11) | 10.68 (0.11) | 15.56 (0.12) | 16.53 (0.32) | < 0.0001 |
| **Fruits juice** | 3.56 (5.14) | 3.84 (0.07) | 2.63 (0.05) | 5.04 (0.05) | 2.42 (0.06) | 4.72 (0.16) | < 0.0001 |
| **Seafood** | 8.77 (7.37) | 7.81 (0.10) | 8.47 (0.08) | 7.36 (0.07) | 12.33 (0.08) | 2.3 (0.22) | < 0.0001 |
| **Eggs** | 0.76 (0.83) | 0.72 (0.01) | 0.65 (0.00) | 0.68 (0.00) | 1.04 (0.01) | 0.49 (0.02) | < 0.0001 |
| **Milk** | 0.73 (1.80) | 3.25 (0.02) | 0.18 (0.01) | 0.27 (0.01) | 0.18 (0.01) | 0.1 (0.04) | < 0.0001 |
| **Wholegrain products** | 3.23 (4.29) | 2.73 (0.05) | 1.66 (0.04) | 2.81 (0.04) | 5.48 (0.04) | 5.2 (0.13) | < 0.0001 |
| **Legumes** | 0.68 (1.41) | 0.43 (0.01) | 0.42 (0.01) | 0.49 (0.01) | 0.85 (0.01) | 4.25 (0.04) | < 0.0001 |
| **Oil** | 1.39 (1.28) | 1.15 (0.01) | 1.19 (0.01) | 1.34 (0.01) | 1.72 (0.01) | 2.14 (0.04) | < 0.0001 |
| **Fast food** | 3.07 (3.01) | 3.04 (0.04) | 2.79 (0.03) | 4.12 (0.03) | 2.2 (0.03) | 2.72 (0.09) | < 0.0001 |
| **Dressing** | 0.53 (0.65) | 0.51 (0.00) | 0.56 (0.00) | 0.62 (0.00) | 0.43 (0.00) | 0.38 (0.02) | < 0.0001 |

Abbreviations: SFF, Sweetened and Fatty Foods.

^1^Values are mean (SD) for the whole sample, and energy-adjusted means of food budget coefficients (SEM) across clusters (ANCOVA model).

^2^P values were calculated using ANCOVA.

## Supplemental Table 4: Organic food budget coefficients (%) across clusters^1,2^

| **Food groups** | **Whole sample** | **Milk-based** | **Meat-based** | **Fast-food-based** | **Healthy-fish-based** | **Healthy-plant-based** | **p** |
| --- | --- | --- | --- | --- | --- | --- | --- |
| **Whole diet** | 28.71 (26.54) | 21.15 (0.35) | 20.4 (0.29) | 25.83 (0.27) | 40.49 (0.29) | 69.94 (0.79) | < 0.0001 |
| **Vegetables** | 15.52 (16.53) | 14.3 (0.24) | 14.49 (0.19) | 14.52 (0.18) | 17.83 (0.20) | 21.49 (0.54) | < 0.0001 |
| **Nuts** | 2.58 (7.55) | 1.95 (0.11) | 1.64 (0.09) | 2.07 (0.08) | 4.06 (0.09) | 6.65 (0.24) | < 0.0001 |
| **Dairy** | 7.05 (11.23) | 6.19 (0.16) | 6.41 (0.13) | 8.85 (0.12) | 6.82 (0.14) | 2.47 (0.36) | < 0.0001 |
| **Poultry** | 3.95 (8.91) | 3.99 (0.13) | 5.48 (0.1) | 3.66 (0.1) | 3.12 (0.11) | 0.41 (0.29) | < 0.0001 |
| **Meat** | 5.8 (10.75) | 5.63 (0.15) | 9.05 (0.12) | 4.81 (0.12) | 4.38 (0.13) | 0.37 (0.34) | < 0.0001 |
| **Processed meat** | 1.89 (4.86) | 1.72 (0.07) | 2.48 (0.06) | 1.89 (0.05) | 1.6 (0.06) | 0.22 (0.16) | < 0.0001 |
| **Potatoes** | 0.71 (3.17) | 0.77 (0.05) | 0.92 (0.04) | 0.65 (0.04) | 0.54 (0.04) | 0.56 (0.10) | < 0.0001 |
| **Cereals** | 5.28 (10.69) | 5.18 (0.16) | 5.47 (0.13) | 5.92 (0.12) | 4.22 (0.13) | 6.44 (0.35) | < 0.0001 |
| **Beverages** | 5.12 (10.24) | 5.24 (0.15) | 4.67 (0.12) | 5.5 (0.11) | 5.08 (0.12) | 5.04 (0.33) | < 0.0001 |
| **Alcohol** | 2.82 (8.21) | 2.4 (0.12) | 3.25 (0.1) | 2.92 (0.09) | 2.68 (0.10) | 1.58 (0.27) | < 0.0001 |
| **Soya-based food** | 2.53 (8.46) | 1.39 (0.12) | 1.25 (0.1) | 1.84 (0.09) | 3.68 (0.10) | 15.74 (0.26) | < 0.0001 |
| **Fat** | 0.23 (2.58) | 0.2 (0.04) | 0.25 (0.03) | 0.24 (0.03) | 0.24 (0.03) | 0.28 (0.08) | < 0.0001 |
| **SFF** | 6.23 (11.15) | 6.87 (0.16) | 6.14 (0.13) | 7 (0.12) | 5.17 (0.14) | 4.69 (0.36) | < 0.0001 |
| **Fruits** | 11.9 (14.32) | 11.25 (0.21) | 10.69 (0.17) | 11.36 (0.16) | 14.03 (0.17) | 13.64 (0.47) | < 0.0001 |
| **Fruits juice** | 3.89 (9.23) | 4.16 (0.13) | 3.28 (0.11) | 5.36 (0.1) | 2.58 (0.11) | 4.07 (0.30) | < 0.0001 |
| **Seafood** | 5.13 (10.32) | 4.4 (0.15) | 5.31 (0.12) | 4.55 (0.11) | 6.68 (0.13) | 1.21 (0.34) | < 0.0001 |
| **Eggs** | 2.86 (9.81) | 3.09 (0.14) | 3.01 (0.12) | 2.97 (0.11) | 2.71 (0.12) | 0.58 (0.32) | < 0.0001 |
| **Milk** | 0.92 (5.06) | 3.89 (0.07) | 0.29 (0.06) | 0.43 (0.05) | 0.23 (0.06) | 0.12 (0.16) | < 0.0001 |
| **Wholegrain products** | 3.98 (9.03) | 3.38 (0.13) | 2.35 (0.11) | 3.56 (0.1) | 6.45 (0.11) | 5.15 (0.29) | < 0.0001 |
| **Legumes** | 0.77 (2.38) | 0.5 (0.03) | 0.5 (0.03) | 0.59 (0.03) | 0.99 (0.03) | 4.35 (0.07) | < 0.0001 |
| **Oil** | 2.22 (7.17) | 2.05 (0.1) | 2.17 (0.08) | 2.2 (0.08) | 2.41 (0.09) | 2.44 (0.23) | < 0.0001 |
| **Fast food** | 1.58 (4.39) | 1.35 (0.06) | 1.37 (0.05) | 2.13 (0.05) | 1.27 (0.05) | 1.89 (0.14) | < 0.0001 |
| **Dressing** | 0.47 (3.59) | 0.48 (0.05) | 0.54 (0.04) | 0.53 (0.04) | 0.35 (0.04) | 0.3 (0.12) | < 0.0001 |

Abbreviations: SFF, Sweetened and Fatty Foods.

^1^ Values are mean (SD) for the whole sample, and energy-adjusted means (SEM) of the organic food budget coefficients across clusters (ANCOVA model). Conventional food budget coefficients are the complementary values to 100%.

^2^ P values were calculated using ANCOVA.

## Supplemental Table 5: Budget coefficients of protein intake (%) across clusters^1,2^

| **Food groups** | **Whole sample** | **Milk-based** | **Meat-based** | **Fast-food-based** | **Healthy-fish-based** | | **Healthy-plant-based** | **p** |
| --- | --- | --- | --- | --- | --- | --- | --- | --- |
| **Whole diet** | 9.31 (2.91) | 9.07 (0.03) | 10.72 (0.03) | 8.78 (0.03) | 9.16 (0.03) | 5.46 (0.08) | | < 0.0001 |
| **Vegetables** | 0.19 (0.12) | 0.16 (0.00) | 0.16 (0.00) | 0.17 (0.00) | 0.23 (0.00) | 0.33 (0.00) | | < 0.0001 |
| **Nuts** | 0.29 (0.54) | 0.18 (0.00) | 0.13 (0.00) | 0.21 (0.00) | 0.52 (0.00) | 1.1 (0.01) | | < 0.0001 |
| **Dairy** | 1.1 (1.08) | 0.94 (0.01) | 0.94 (0.01) | 1.56 (0.01) | 0.93 (0.01) | 0.43 (0.03) | | < 0.0001 |
| **Poultry** | 0.77 (0.74) | 0.77 (0.01) | 1.13 (0.00) | 0.62 (0.00) | 0.67 (0.00) | 0.08 (0.02) | | < 0.0001 |
| **Meat** | 2.66 (2.08) | 2.84 (0.02) | 4.24 (0.02) | 2.06 (0.01) | 1.93 (0.02) | 0.19 (0.05) | | < 0.0001 |
| **Processed meat** | 0.67 (0.62) | 0.69 (0.00) | 0.88 (0.00) | 0.69 (0.00) | 0.5 (0.00) | 0.07 (0.01) | | < 0.0001 |
| **Potatoes** | 0.01 (0.01) | 0.01 (0.00) | 0.01 (0.00) | 0.01 (0.00) | 0.01 (0.00) | 0.01 (0.00) | | < 0.0001 |
| **Cereals** | 0.34 (0.26) | 0.38 (0.00) | 0.35 (0.00) | 0.4 (0.00) | 0.22 (0.00) | 0.37 (0.00) | | < 0.0001 |
| **Beverages** | 0.02 (0.03) | 0.06 (0.00) | 0.01 (0.00) | 0.01 (0.00) | 0.01 (0.00) | 0 (0.00) | | < 0.0001 |
| **Alcohol** | 0 (0.01) | 0 (0.00) | 0.01 (0.00) | 0 (0.00) | 0 (0.00) | 0 (0.00) | | < 0.0001 |
| **Soya-based food** | 0.08 (0.27) | 0.03 (0.00) | 0.02 (0.00) | 0.05 (0.00) | 0.12 (0.00) | 0.76 (0.00) | | < 0.0001 |
| **Fat** | 0 (0.00) | 0 (0.00) | 0 (0.00) | 0 (0.00) | 0 (0.00) | 0 (0.00) | | < 0.0001 |
| **SFF** | 0.35 (0.28) | 0.38 (0.00) | 0.32 (0.00) | 0.44 (0.00) | 0.27 (0.00) | 0.34 (0.00) | | < 0.0001 |
| **Fruits** | 0.08 (0.08) | 0.07 (0.00) | 0.07 (0.00) | 0.07 (0.00) | 0.11 (0.00) | 0.11 (0.00) | | < 0.0001 |
| **Fruits juice** | 0.02 (0.03) | 0.02 (0.00) | 0.01 (0.00) | 0.03 (0.00) | 0.01 (0.00) | 0.02 (0.00) | | < 0.0001 |
| **Seafood** | 1.86 (1.59) | 1.65 (0.02) | 1.79 (0.01) | 1.56 (0.01) | 2.64 (0.01) | 0.49 (0.04) | | < 0.0001 |
| **Eggs** | 0.09 (0.10) | 0.08 (0.00) | 0.08 (0.00) | 0.08 (0.00) | 0.12 (0.00) | 0.06 (0.00) | | < 0.0001 |
| **Milk** | 0.02 (0.06) | 0.12 (0.00) | 0 (0.00) | 0.01 (0.00) | 0 (0.00) | 0 (0.00) | | < 0.0001 |
| **Wholegrain products** | 0.28 (0.39) | 0.24 (0.00) | 0.14 (0.00) | 0.25 (0.00) | 0.48 (0.00) | 0.38 (0.01) | | < 0.0001 |
| **Legumes** | 0.05 (0.10) | 0.03 (0.00) | 0.03 (0.00) | 0.03 (0.00) | 0.06 (0.00) | 0.31 (0.00) | | < 0.0001 |
| **Oil** | 0 (0.00) | 0 (0.00) | 0 (0.00) | 0 (0.00) | 0 (0.00) | 0 (0.00) | | < 0.0001 |
| **Fast food** | 0.32 (0.32) | 0.31 (0.00) | 0.29 (0.00) | 0.43 (0.00) | 0.22 (0.00) | 0.27 (0.01) | | < 0.0001 |
| **Dressing** | 0.01 (0.01) | 0.01 (0.00) | 0.01 (0.00) | 0.01 (0.00) | 0.01 (0.00) | 0.01 (0.00) | | < 0.0001 |

Abbreviations: SFF, Sweetened and Fatty Foods.

^1^ Values are mean (SD) for the whole sample, and energy-adjusted means of budget coefficients of protein intake (SEM) across clusters (ANCOVA model).

^2^ P values were calculated using ANCOVA.

## Supplemental Figure 1: Selection of the study sample

n=37,685 had completed the organic food questionnaire between June and December 2014

n=37,305 had no missing covariates

n=35,196 were not under-reporter or over-reporter

n=34,453 were not living overseas

n=29,210 had available data regarding the place of purchase

FINAL SAMPLE n=29,210

## Supplemental Figure 2: Food group consumption across clusters (g/d)^1^


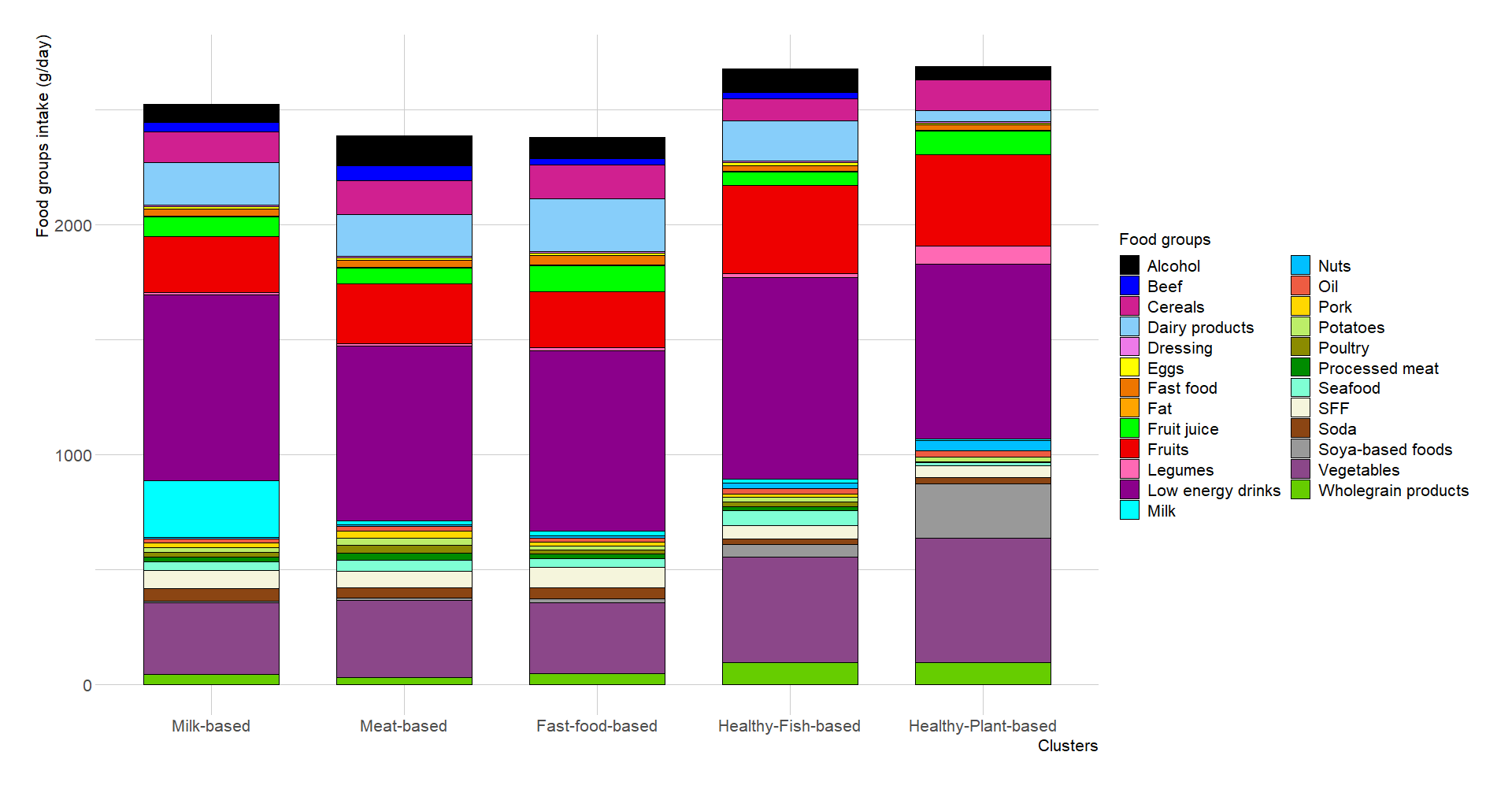


^1^ Values are energy-adjusted means of food groups intake (g/d) computed using ANCOVA model. Food groups are formed as follows: Vegetables include all vegetables and soups; Fruits include fresh fruits, fruits in syrup and compote, dried fruits and seeds; Low energy drinks include all non-alcoholic beverages that are fruit nectar, syrup, plant-based beverages (except soya-based), milk consumed with tea/coffee; Dairy products include yogurts, fresh cheese and cheese; Potatoes include other tubers; Cereals include breakfast cereal low in sugar, bread semolina, rice and pasta; SFF (sweet and fat foods) include croissants, pastries, chocolate, biscuits, milky dessert, ice cream, honey and marmalade, cakes, chips, salted oilseeds, salted biscuits; Fast-food include sandwich, prepared foods such as pizza, hamburger, ravioli, panini, salted pancake, etc.; Soya-based food includes that are tofu, soya-based meat substitute and vegetable patties, soya-based yogurt, soya-based milk; Fat includes animal fat (butter and lards); and Dressing includes ready-to-use salad dressing, mayonnaise or cream-based sauces sour cream and butter and all fat-based sauces; Oil includes plant-based oils.

1. Pointereau P, Langevin B, Gimaret M. DIALECTE, a comprehensive and quick tool to assess the agro-environmental performance of farms. Prod Reprod Farming Syst New Modes Organ Sustain Food Syst Tomorrow 10th Eur IFSA Symp Aarhus Den 1-4 July 2012 [Internet]. 2012 [cité 14 mars 2023]; Disponible sur: https://www.cabdirect.org/cabdirect/abstract/20133410218

2. Baudry J, Pointereau P, Seconda L, Vidal R, Taupier-Letage B, Langevin B, et al. Improvement of diet sustainability with increased level of organic food in the diet: findings from the BioNutriNet cohort. Am J Clin Nutr. avr 2019;109(4):1173‑88.

3. Seconda L, Baudry J, Allès B, Boizot-Szantai C, Soler LG, Galan P, et al. Comparing nutritional, economic, and environmental performances of diets according to their levels of greenhouse gas emissions. Clim Change. mai 2018;148(1‑2):155‑72.

4. Kramer GF, Tyszler M, Veer P van’t, Blonk H. Decreasing the overall environmental impact of the Dutch diet: how to find healthy and sustainable diets with limited changes. Public Health Nutr. juin 2017;20(9):1699‑709.

5. Goedkoop M, Heijungs R, Huijbregts M. ReCiPE 2008: A life cycle impact assessment method which comprises harmonised category indicators at the midpoint and the endpoint level. 2013;

6. de Gavelle E, Huneau JF, Fouillet H, Mariotti F. The Initial Dietary Pattern Should Be Considered when Changing Protein Food Portion Sizes to Increase Nutrient Adequacy in French Adults. J Nutr. mars 2019;149(3):488‑96.

7. Verger EO, Mariotti F, Holmes BA, Paineau D, Huneau JF. Evaluation of a Diet Quality Index Based on the Probability of Adequate Nutrient Intake (PANDiet) Using National French and US Dietary Surveys. Cameron DW, éditeur. PLoS ONE. 3 août 2012;7(8):e42155.

8. Kesse-Guyot E, Chaltiel D, Fezeu LK, Baudry J, Druesne-Pecollo N, Galan P, et al. Association between adherence to the French dietary guidelines and the risk of type 2 diabetes. Nutrition. avr 2021;84:111107.

9. Chaltiel D, Adjibade M, Deschamps V, Touvier M, Hercberg S, Julia C, et al. Programme National Nutrition Santé – guidelines score 2 (PNNS-GS2): development and validation of a diet quality score reflecting the 2017 French dietary guidelines. Br J Nutr. août 2019;122(03):331‑42.

10. Keaver L, Ruan M, Chen F, Du M, Ding C, Wang J, et al. Plant- and animal-based diet quality and mortality among US adults: a cohort study. Br J Nutr. 28 juin 2021;125(12):1405‑15.

11. Murray CJ, Ezzati M, Flaxman AD, Lim S, Lozano R, Michaud C, et al. GBD 2010: design, definitions, and metrics. The Lancet. déc 2012;380(9859):2063‑6.

12. Fouillet H, Dussiot A, Perraud E, Wang J, Huneau JF, Kesse-Guyot E, et al. Plant to animal protein ratio in the diet: nutrient adequacy, long-term health and environmental pressure [Internet]. Nutrition; 2022 mai [cité 20 mars 2023]. Disponible sur: http://medrxiv.org/lookup/doi/10.1101/2022.05.20.22275349

13. Murray CJL, Aravkin AY, Zheng P, Abbafati C, Abbas KM, Abbasi-Kangevari M, et al. Global burden of 87 risk factors in 204 countries and territories, 1990–2019: a systematic analysis for the Global Burden of Disease Study 2019. The Lancet. oct 2020;396(10258):1223‑49.

14. INSEE. Définition - Nombre d’unités de Consommation Du Ménage Fiscal | Insee. https://www.insee.fr/fr/metadonnees/definition/c1001. 2009.

15. Kantar. Kantar | Consumer Panel | Consumer Behaviour Insights | Consumer Panels - Kantar Worldpanel. https://www.kantarworldpanel.com/global. 2012.

16. FactoMineR.pdf [Internet]. [cité 21 sept 2023]. Disponible sur: https://cran.r-project.org/web/packages/FactoMineR/FactoMineR.pdf
